# Nitrogen deficiency impacts growth and modulates carbon metabolism in maize

**DOI:** 10.1007/s00425-025-04814-x

**Published:** 2025-09-02

**Authors:** Joseph N. Amoah, Claudia Keitel, Brent N. Kaiser

**Affiliations:** School of Life and Environmental Sciences, University of Sydney, 380 Werombi Road, Brownlow Hill, Camden, NSW 2570 Australia

**Keywords:** Carbon partitioning and accumulation, Low nitrogen, Root-to-shoot ratio, Sink–source dynamics, Sugar and starch metabolism

## Abstract

**Main conclusion:**

Nitrogen (N) deficiency in maize regulates carbon (C) metabolism by enhancing sugar and starch metabolism and related gene expression in both shoots and roots, while increasing root competition for assimilates causing carbohydrate accumulation in leaves and sheaths due reduced translocation to sink tissues.

**Abstract:**

Soluble sugars are vital for plant development, with nitrogen (N) availability playing a key role in their distribution across plant organs, ultimately shaping growth patterns. However, the regulatory mechanisms governing carbon (C) assimilate allocation and utilization under different N forms remain unclear. This study examined C fixation, utilization, and spatial distribution in hydroponically grown maize seedlings subjected to four N treatments: 1 mM NO_3_⁻ (low N, LN), 2 mM NO_3_⁻ (medium N), 10 mM NO_3_⁻ (high N), and 1 mM NH_4_⁺ (low ammonium, LA). LN treatment significantly increased soluble sugar and starch contents while promoting greater root biomass at the expense of shoot biomass, leading to a higher root-to-shoot assimilate allocation. The activities of sugar and starch metabolism enzymes were more tightly regulated in both shoots and roots under LN, indicating enhanced C utilization and increased competition for assimilates, particularly in the root. Key genes involved in above-ground sugar and starch metabolism, *ZmSPS1*, *ZmSuSy1*, *ZmCINV1*, *ZmVINV1*, *ZmCWINV1*, *ZmSTP2*, *ZmSUC2*, *ZmSWEET14*, *ZmSS1*, *ZmAMY1*, *ZmBAM1*, and *ZmAGPase1*, were upregulated under LN, correlating with enhanced enzyme activity and resulting increased sugar and starch accumulation. Starch and sucrose accumulated more in LN-treated leaves than in other N treatments, with starch primarily stored in leaf tips and sucrose concentrated in the leaf sheath. This pattern suggests that excess C accumulation results from inefficient C utilization in sink tissues rather than impaired C assimilation. These findings provide new insights into how LN modulates C partitioning between leaves and roots for stress adaptation, highlighting the importance of improving C utilization in sink tissues to mitigate N deficiency and enhance plant growth.

**Supplementary Information:**

The online version contains supplementary material available at 10.1007/s00425-025-04814-x.

## Introduction

Nitrogen (N) is an essential nutrient for plant growth and development, serving as a fundamental component of biomolecules such as nucleic acids, amino acids, and proteins. It also serves as a crucial signaling molecule, influencing numerous plant processes, including lateral root growth, resistance to biotic and abiotic stresses, regulation of seed germination, and mediation of hormone signaling (Ahmad et al. 2014). Due to its susceptibility to adsorption, leaching, and transformation in the soil, N availability is often a major limiting factor for crop productivity. Hence growers apply large quantities of N fertilizers to mitigate N limitations. However, less than 50% of the applied N is effectively utilized by crops, with the remainder lost due to excessive rainfall or irrigation, which leads to significant nitrate leaching from the soil profile (Govindasamy et al. 2023). This loss creates a fluctuating N supply characterized by alternating periods of N deficiency, sufficiency, or excess, which can arise in agricultural soils due to environmental factors or as a consequence of agricultural practices. This unpredictable nutrient dynamic negatively impacts various metabolic functions in plants, ultimately hindering growth and productivity (Govindasamy et al. 2023). Thus, understanding crop growth and development under different N treatments, particularly under nitrogen-deficient conditions, is crucial for ensuring food security and promoting sustainable crop production.

N forms differentially regulate N metabolism, affecting protein and amino acid dynamics (Yang et al. 2020). Studies have shown that NO₃⁻ treatment improves N assimilation and stimulates glutamine synthetase (GS)-related gene expression, enhancing amino acid biosynthesis and production of structural and functional proteins (Zhang and Wu 2023). While NO₃⁻ promotes amino acid and protein synthesis, NH₄⁺ treatment triggers distinct catabolic responses by activating proteolytic enzymes, such as cysteine and aspartic proteases. These enzymes accelerate protein degradation, leading to shifts in amino acid composition and redistribution, which reflect a characteristic metabolic adaptation to NH₄⁺ exposure (González-Moro et al. 2021). These proteolytic activities under NH₄⁺ conditions significantly influence C/N interactions. In particular, excessive NH₄⁺ supply can disrupt C balance by diverting C skeletons toward N assimilation and stress-related pathways, thereby impairing carbohydrate metabolism and energy homeostasis (Li et al. 2023). Although protein degradation is a general response to NH₄⁺ presence, the disruption of C balance is more pronounced under high NH₄⁺ concentrations, underscoring the dose-dependent nature of NH₄⁺-induced metabolic reprogramming.

To mitigate the detrimental effects of N-deficiency-induced stress, plants have evolved various adaptive mechanisms, including reduced biomass accumulation and photosynthesis, decreased N uptake, and regulating shoot and root growth (Zhao et al. 2020). Specifically, under LN condition, reduced root biomass and even further reduced shoot biomass resulting in an elevated root-to-shoot (R/S) ratio have been identified as key traits of N-efficient crops (Lopez et al. 2023). While some insights have been gained into the molecular mechanisms regulating root growth in response to LN (Lai et al. 2023), these studies have primarily focused on root architecture without addressing C allocation between the root and shoot under different N forms, particularly under LN conditions. Importantly, root response is closely linked to the supply of assimilates, which not only serve as source of energy but also act as signaling molecules and enhance root adaptation to LN stress. Hence, numerous studies have observed C accumulation in roots under LN conditions (Zhao et al. 2020). However, the regulation of assimilate partitioning between root and shoot under LN is poorly understood. The critical initial step in assimilate utilization is the breakdown of sucrose, after long-distance transport from source to sink into hexoses by sucrose synthase or invertase (Lemoine et al. 2013). Interestingly, elevated root invertase activity has been associated with a higher R/S ratio in soybean under drought stress (Du et al. 2020a), suggesting a link between sucrose metabolism and root growth under stress conditions. In this context, we hypothesize that sucrose-degrading enzymes play a crucial role in regulating assimilate allocation between root and shoot under LN conditions.

N deficiency reduces photosynthesis; however, studies have shown that sugars and starch accumulate in leaves under LN conditions despite a decline in the photosynthetic rate, a response that appears to be conserved across many plant species (Mu and Chen 2021). To date, the mechanism underlying LN-induced C accumulation in leaves remains unclear. Research suggests that LN-reduced sink demand limits the translocation of photo-assimilates, despite enhanced export capacity from source leaves. Consequently, excess C accumulates in source tissues such as leaves and sheaths, primarily due to the imbalance between C supply through photosynthesis and its limited utilization by sink organs (Huang et al. 2022). Given the contradictory findings on C accumulation in source and sink tissues, it is crucial to elucidate the physiological basis of N-deficiency-induced C accumulation. Similar to modulating the activities of C metabolism in sink tissue, sucrose and starch metabolism enzymes were induced in LN leaves, demonstrating an increase in starch and sucrose synthesis, that improved C accumulation. Conversely, sucrose synthase activity in root tissues has been shown to increase under LN conditions, particularly in the elongation and maturation zones, where it supports root growth by facilitating sucrose cleavage and promoting C partitioning to structural and storage compounds (Zhao et al. 2020). This enhanced activity contributes to increased starch accumulation and localized utilization of sugars in roots, aiding in root expansion under N-limited conditions. The C pool in plant leaves is determined by the balance between C fixation, which generally follows a diurnal rhythm, making the C pool dynamic throughout the day (Amoah and Kaiser 2025). Hence, examining the diurnal fluctuations and spatial distribution of assimilates can provide valuable insights into the mechanisms driving LN-induced C accumulation in leaves.

Maize (*Zea mays* L.) was chosen for this study due to its significance as a staple food crop and its role as an ideal model for understanding N metabolism and C allocation. Its well-documented growth patterns, responsiveness to environmental factors, and ability to adapt to various N conditions make it an excellent crop for studying the effects of N form substitution on C distribution (Dong et al. 2023). Furthermore, the economic importance of maize in global agriculture highlights the potential impact of optimizing nutrient management to enhance crop productivity. This study aims to unravel the mechanism underpinning C allocation between the shoot and root of maize. To achieve this, we investigated the effects of different N (mainly NO_3_^−^ and NH_4_^+^) forms on the activities of sucrose metabolism enzymes involved in sucrose utilization within the shoot and root sinks of the maize inbred line TX-40 J and their relationship with C accumulation during photosynthesis. We examined the diurnal fluctuations of assimilates in source leaves and their distribution across various maize tissues, including upper and middle leaves, leaf sheaths, and roots, during both vegetative and reproductive growth stages under different N treatments. In addition, the total protein and amino acid content was quantified to understand N assimilation and remobilization under different N treatments. The findings of this study will enhance our understanding of how N forms regulate C partitioning and serve as a valuable reference for optimizing crop production under varying N conditions, particularly under N deficiency conditions.

## Materials and methods

### Plant materials and experimental site

Seeds of the fast-flowering, short-cycle inbred mini-maize line TX-40 J were used in this study (Amoah and Kaiser 2025). This maize line offers significant advantages, including a uniform genetic background, a shorter lifecycle, and early flowering, which enable efficient and targeted research in various areas of plant biology. The traits observed in this variety serve as valuable references for breeders, facilitating the development of hybrids with enhanced performance and improved nitrogen use efficiency (McCaw et al. 2021). The seeds were germinated in Oasis Horticube Propagation Slabs (Aqua Gardening, Brisbane, Australia) placed in germination trays. The trays were transferred to a climate-controlled growth room, set to a 14/10 day–night cycle, with temperatures of 25 °C during the day and 22 °C at night, and 80% relative humidity for 5 d to allow for seed germination. Once the seedlings had germinated uniformly, they were transferred to a temperature-controlled glasshouse for the subsequent experiments (Amoah and Kaiser 2025).

### Experimental treatment, set up, and sampling

Seedlings were divided into four treatment groups and cultivated in 3-L pots, with their roots supported by inorganic expanded clay pellets (Aqua Gardening, Brisbane, Australia). Each group received a specific nitrogen (N) source: 1 mM NO₃⁻ (low N; LN), 2 mM NO₃⁻ (medium N; MN), 10 mM NO₃⁻ (high N; HN), and 1 mM NH₄⁺ (low NH₄⁺; LA). These nutrient levels were selected based on previous studies (Amoah and Kaiser 2025). The seedlings were grown for 40 days.

The system was set up in a climate-controlled glasshouse, with conditions matching those of the growth room used for seed germination, but with supplemental LED lighting providing 1000 µmol m⁻^2^ s⁻^1^ at pot level. Each system was designed to accommodate 40 pots, with one plant per pot. Plants were drip-irrigated with the respective nutrient solution, which was circulated through a hydroponic pump system. Irrigation occurred twice daily for 1 min, at 12:00 PM and 5:00 PM. The nutrient solution comprised the following concentrations (in mM): 1.0 MgSO₄, 1.0 KH₂PO₄, 0.05 H₃BO₃, 0.005 MnSO₄, 0.001 ZnSO₄, 0.001 CuSO₄, 0.001 Na₂MoO₄, 0.1 KCl, 0.1 Fe-EDTA, 0.1 Fe-EDDHA, 2.5 Ca(NO₃)₂, 0.25 K₂SO₄, 0.25 CaCl₂, and 1.75 CaSO₄ (Table S1) and were stored in 162-L Brute containers with lids (Rubbermaid, Atlanta, GA, USA). The solution was changed weekly, with daily pH adjustments with 1 M H_2_SO_4_ or 1 M NaOH to maintain a stable pH of 5.9. The treatment solution was delivered to the system by an Eden 140G FL submersible water pump (Creative Pumps, Beverley, Australia). Plants were uniquely identified and randomized into four blocks using the ‘agricolae’ package of the R statistical software (v4.4.2).

Sampling was conducted at 20 (vegetative, V6 stage) and 40 (reproductive, R1 stage) days after treatment (DAT). Fresh leaf, root, and ear tissues for biochemical analysis were collected, immediately frozen in liquid nitrogen (N₂), and stored at − 80 °C. Shoot and root samples for biomass analysis were oven-dried at 70 °C for 48 h to determine dry weights. The shoot and root biomass values were summed to calculate the total plant biomass in grams (g DW).

### Spatial distribution and diurnal changes determination

To examine the spatial distribution of sugars and starch, the youngest emerging leaf at 20 and 40 DAT was sampled and divided into three equal segments. Additional samples included the corresponding leaf sheath, root, basal 5 cm of expanded leaves, root, and ear. Sampling was conducted at 22:00 and again at 6:00 the following morning on 20 and 40 DAT. For diel analysis, the middle sections of the youngest fully expanded leaves were collected at 7:00, 12:00, 17:00, 22:00, and 7:00 (the next day) on 20 and 40 DAT. All samples were immediately frozen in liquid N_2_ and stored at − 80 °C for subsequent biochemical analysis. To further investigate the diurnal variations in sugar metabolism under different N forms, the rates of leaf sucrose and starch synthesis and degradation and net sucrose and starch accumulation were calculated based on the diurnal sucrose and starch data, using the formulas in Table S2.

### Net photosynthetic rate and chlorophyll and nitrogen (N) measurement

The net photosynthetic rate (Pn) was measured on the young emerging blade (YEB) of each treatment using a portable LI-6400 photosynthetic system (LI-COR Inc., Lincoln, NE, USA). Measurements were taken at 9:00 AM and 11:00 AM. Cuvette conditions included a light level of 1000 µmol m⁻^2^ s⁻^1^, CO₂ concentration of 400 ppm, flow rate of 500 µmol m⁻^2^ s⁻^1^, and relative humidity between 60 and 65%. Chlorophyll pigment was extracted from the youngest fully expanded blade (YEB) following Licor measurements. The YEB samples were harvested and ground into a fine powder, and 0.2 g of the ground tissue was extracted with 100% methanol on a shaker at 25 °C until complete bleaching occurred (Amoah et al. 2019). The extract was then centrifuged at 10,000 g for 10 min, and the absorbance of the resulting supernatant was measured at 470, 646, and 663 nm using a UV–Vis spectrophotometer (Shimadzu, Tokyo, Japan). Chlorophyll concentration was calculated based on the equations described by Amoah and Kaiser (2025).

N content was determined using the Kjeldahl method as described by Rizvi et al. (2022), with minor modifications. A 0.2 g sample of YEB was digested with 0.5 mL of concentrated H₂SO₄ and 0.5 mL of a catalyst mixture consisting of 10 g of K₂SO₄ and 1 g of CuSO₄. The mixture was heated at 100 °C for 60 min on a heating block in a fume hood. After digestion, the samples were allowed to cool, and 0.5 mL of 40% NaOH solution was slowly added, followed by 0.5 mL of distilled water. Subsequently, 1 mL of the resulting mixture was combined with 1 mL of Nessler’s reagent and incubated for 10 min at room temperature. The absorbance was measured at 420 nm using a UV–Vis spectrophotometer (Shimadzu). Total N content was determined from a standard curve generated with (NH₄)₂SO₄ standards.

### Soluble sugar, starch, glucose, and sucrose content determination

Soluble sugar and sucrose content were measured as described by Amoah and Adu-Gyamfi (2024). Briefly, 100 mg of ground samples were homogenized in 1 mL of 80% (v/v) ethanol, and the mixture was heated at 80 °C for 30 min. After cooling for 5 min, the mixture was centrifuged at 12,000 g for 10 min. The supernatants were collected, and total soluble sugars were determined using the anthrone reagent, with absorbance measured at 620 nm. Sucrose content was determined by the resorcinol-HCl method, which forms a pink complex measured at 480 nm using a UV–Vis spectrophotometer (Shimadzu). The ethanol-insoluble residue was used for starch extraction following the procedure outlined by Du et al. (2020a). After evaporating the ethanol, 1 mL of distilled water was added to the samples, which were then incubated at 100 °C for 15 min. Starch was hydrolyzed using separate treatment of 9.2 M perchloric acid (HClO₄). The resulting glucose units were quantified using the anthrone reagent, and absorbance was measured at 620 nm using a UV–Vis spectrophotometer (Shimadzu). Glucose and fructose content were determined using the anthrone colorimetry method as described by Dong et al. (2023). A mixture of 1 mL of supernatant and 5 mL of anthrone diluted sulfuric acid reagent was boiled for 10 min. A blank was prepared similarly, using 1 mL of distilled Milli-Q water instead of the supernatant. After cooling, the solution’s absorbance was measured at 620 nm using a spectrophotometer, with the blank adjusted to zero. For fructose content, 1 mL of extract, 1 mL of 0.1% (v/v) hydroquinone, and 3.5 mL of 30% (v/v) HCl were combined in a test tube, thoroughly mixed, and heated at 80 °C for 10 min in a water bath. After cooling, the solution’s absorbance was measured at 480 nm using a spectrophotometer, with the blank adjusted to zero. The measured absorbance was used to calculate fructose content based on a standard curve.

### Sugar metabolism enzymes activity assays

Enzymes were extracted following the method described by Chen et al. (2019), with slight modifications. A 100 mg plant tissue sample was ground to a fine powder using liquid N_2_ and homogenized in 1 mL of ice-cold extraction buffer. The buffer contained 50 mM HEPES–NaOH (pH 7.5), 5 mM MgCl₂, 0.1% (v/v) β-mercaptoethanol, 0.05% (v/v) Triton-X100, 0.05% (w/v) BSA, 2% (w/v) polyvinylpyrrolidone, and 1 mM EDTA. The homogenate was centrifuged at 12,000 g for 10 min at 4 °C, and the supernatant was used for the assays of sucrose synthase (SuSy), vacuolar invertase (VINV), and cytoplasmic invertase (CINV) activities. The pellet was washed twice with 0.5 mL of extraction buffer and then resuspended in 1.8 mL of salt extraction buffer. Samples were extracted overnight at 4 °C and centrifuged at 12,000 g for 10 min at 4 °C. The resulting supernatant was used for the assay of cell wall invertase activity (CWINV).

SuSy and CINV activities were determined following the method by Li et al. (2021), while VINV activity was assayed according to Chen et al. (2019). For SuSy activity, 100 μL of enzyme extract was mixed on ice with 200 μL of a reaction solution containing 80 mM MES-NaOH (pH 5.5), 5 mM NaF, 100 mM sucrose, and 5 mM UDP. The reaction was incubated at 30 °C for 30 min and terminated by boiling for 2 min. A control reaction lacking UDP was included. For VINN, CINV, and CWIN assays, the procedure was similar, but with variations in the reaction mixtures. VIN activity was measured using 200 mM acetic acid-sodium acetate buffer (pH 4.5 or 4.8) with 100 mM sucrose. CINV activity utilized 100 mM HEPES–NaOH buffer (pH 7.5) with 100 mM sucrose. For sucrose phosphate synthase (SPS) activity measurement, 0.1 g of frozen tissue was homogenized in an extraction buffer containing 50 mM Tris–HCl (pH 7.5), 1 mM EDTA, 1 mM MgCl_2_, 12.5% (v/v) glycerine, 10% polyvinylpyrrolidone (PVP), and 10 mM mercaptoethanol. For SPS activity, 200 μL of supernatant was mixed with reaction buffer containing 200 mM Tris–HCl (pH 7.0), 40 mM MgCl_2_, 12 mM UDP-glucose, 40 mM fructose-6-P, and 200 μL extract. Another reaction buffer containing 12 mM UDP, 40 mM sucrose, 200 mM Tris–HCl (pH 7.0), and 40 mM MgCl_2_ was prepared. The mixture was incubated at 30 °C for 30 min and terminated using 100 μL of 2 M of NaOH. The mixture was incubated at 100 °C for 10 min to destroy untreated hexose and hexose phosphates, cooled to room temperature, and mixed with 1 mL of 0.1% (w/v) resorcin in 95% (v/v) ethanol and 3.5 mL of 30% (w/v) HCl. The solution was incubated for 10 min at 80 °C. Sucrose content in the SPS reaction was calculated using a standard curve measured at A480 nm using anthrone reagent.

### Starch metabolism enzymes activity assay

For starch synthase (SS) activity, 100 mg of tissue samples were homogenized in an extraction buffer containing 50 mM Tris–HCl (pH 7.0), 10% glycerol, 10 mM EDTA, 5 mM DTT, 1 mM PMSF, and 50 μL/g tissue of 10 × Protease Inhibitor Cocktail (Sigma-Aldrich, Cat# P9599). The homogenate was centrifuged at 12,000 g for 10 min, and the supernatant was collected. A reaction mixture was prepared by mixing 0.1 mL of the supernatant with 0.9 mL of a solution containing 50 mM Tris–HCl (pH 7.0), 5 mM ADP-glucose, 1 mg/mL glycogen, and 10 mM MgCl₂. The reaction mixture was incubated at 30 °C for 30 min. To stop the reaction, 0.1 M HCl was added to denature the enzymes. To detect inorganic phosphate (Pi) consumption, 1% (w/v) ammonium molybdate was added. The mixture was incubated at room temperature for 30 min, and the absorbance was recorded at a wavelength of 620 nm using a UV–Vis spectrophotometer (Shimadzu). A standard curve was prepared using known Pi concentrations, and starch synthase activity was calculated as the amount of Pi released, expressed in μmol g⁻^1^FW.

The activities of α-and β-amylase were measured according to the methods by Du et al. (2020a, b) with minor modifications. Briefly, tissue samples were homogenized in 1 mL of chilled distilled water and centrifuged at 12,000 g for 15 min. The supernatants were separated and used for quantifying α-and β-amylase. For α-amylase activity, 0.5 mL of supernatant was mixed with 3 mM CaCl_2_, heated at 70 °C for 5 min to inactivate β-amylase, cooled to room temperature, followed by the addition of 2% starch solution in 0.1 M citrate buffer. The mixture was incubated at 30 °C for 5 min and stopped by adding 1 mL of color reagent dinitrosalicylic acid (DNS). The mixture was heated at 50 °C for 5 min, cooled down, and the α-amylase activity was determined by recording absorbance at 540 nm wavelength with a UV–Vis spectrophotometer (Shimadzu). β-Amylase activity (BAM) was assayed by initially inactivating α-amylase (AMY) with 0.1 M EDTA. Then a 1 mL solution containing 0.1 M EDTA, 2% starch solution, and 0.1 mM citrate buffer were mixed with 0.5 mL enzyme extract. The mixture was incubated at 30 °C for 5 min. The reaction was stopped by adding 1 mL of color reagent (dinitrosalicylic acid). The β-amylase activity was measured by recording absorbance at 540 nm wavelength with a UV–Vis spectrophotometer (Shimadzu).

ADP-glucose pyrophosphorylase (AGPase) activity was determined using previously described methods by Amoah et al. (2025), with minor modifications. Briefly, 100 mg of fresh samples were homogenized in 1 mL of ice-cold extraction buffer containing 0.1 M Tris–HCl (pH 7.9), 5 mM glutathione, and 1 mM EDTA. The homogenate was centrifuged at 15,000 g for 20 min at 4 °C, and the supernatant was collected. Subsequently, 0.1 mL of the supernatant was mixed with 0.9 mL of a reaction mixture containing 0.4 M Tris–HCl buffer (pH 7.9), 0.06 M MgSO₄, 48 mM cysteine, 2.4 mg/mL BSA, 4 mM ADP-glucose, 20 mM sodium pyrophosphate, 30 mM 3-phosphoglycerate, and 4 units each of glucose-6-phosphate dehydrogenase and phosphoglucomutase. Afterwards, 0.1 mL of enzyme extract was added to NADP⁺ as the final component. The absorbance was measured at 340 nm using a UV–Vis spectrophotometer (Shimadzu). The AGPase activity was expressed as μmol min⁻^1^ g⁻^1^ FW.

### Total amino acid quantification

Total amino acids content was determined using the methods outlined by Bates et al. (1973). Frozen leaf tissues (100 mg) were homogenized in 10 mL of 3% (v/v) aqueous sulfosalicylic acid. After filtering the homogenate, 1 mL of filtrate was combined with 1 mL of glacial acetic acid and 1 mL of acidic ninhydrin. The resulting mixture was incubated at 100 °C for 1 h and then cooled on ice for 20 min before being extracted with 1 mL of toluene. The concentrations of amino acids were measured using a UV–Vis spectrophotometer (Shimadzu) at A580 nm. Leaf and root total protein content was estimated using the Bradford protein assay (Bradford 1976) following manufacturer’s protocol (Bio-Rad, South Granville, Australia).

### RNA isolation, cDNA synthesis, and qPCR analysis

Total RNA was isolated from leaf and root tissues using the TRIzol RNA Isolation Reagents (Invitrogen, Carlsbad, CA, USA) following the manufacturer’s protocol. RNA quantity and integrity were assessed by measuring the optical density at 260 nm and through 1.0% agarose (v/w) gel electrophoresis, respectively. Subsequently, 1 µg of total RNA was reverse-transcribed into single-stranded cDNA using the iScript™ RT Reagent Kit (Bio-Rad, Hercules, CA, USA) according to the manufacturer’s instructions. Quantitative real-time polymerase chain reaction (qPCR) was performed using the CFX 96 Real-Time System (Bio-Rad) with SYBR Green fluorescence (Bio-Rad). The ∆∆CT method was used for data analysis. Gene-specific primers (Table S3) were employed to assess their expression patterns under different N (LN, MN, HN, and LA) treatment conditions. The thermal cycling conditions consisted of an initial denaturation step at 95 °C for 5 min, followed by 40 cycles of 95 °C for 15 s, 55 °C for 15 s, and 72 °C for 30 s. All experiments were conducted with four analytical replicates, and relative transcript levels were normalized using *ZmActin* and *ZmUBQ* as internal controls.

### Statistical analysis

Data were analyzed using a two-way analysis of variance (ANOVA) in R statistical software (v4.4.2), considering growth stage (S) and nitrogen (N) treatment as fixed factors. Tukey’s multiple comparison test was used for post hoc analysis, with significance set at *P* ≤ 0.05. Results are presented as the mean ± standard error (SE) of four biological replicates. Different letters above error bars indicate statistically significant differences. Graphs were generated using GraphPad Prism (v10.4.0), and Pearson’s correlation plots were produced in R (v4.4.2).

## Results

### Plant photosynthesis, total shoot and root biomass, tissue N, total amino acid, and protein contents

The tissue biomass, N, total amino acid, total protein, and leaf net photosynthetic rate (Pn) differed among plants in the different treatment groups. Specifically, at 40 DAT, shoot biomass increased significantly (*P* ≤ 0.05) by 56%, 72%, 66%, and 67% under LN, MN, HN, and LA treatments, respectively, while root biomass rose by 69%, 71%, 47%, and 68% under the same conditions (Table 1). Plants grown under HN treatment exhibited significantly (*P* ≤ 0.05) increased shoot biomass, N content, total amino acids, protein contents, and leaf Pn. However, plants treated with LN showed a significantly (*P* ≤ 0.05) increased root biomass and R/S ratio compared to plants in the other treatment groups (Table 1). Growth stage (S) significantly influenced (*P* ≤ 0.05) shoot and root biomass, as well as leaf and root N, total amino acid, and total protein contents (Table 2). The type and amount of N supplied nitrogen treatment (T) had a significant impact on shoot and root biomass, R/S ratio, and leaf and root total amino acid and protein content. Comparatively, plants grown under NO₃⁻ treatment, particularly MN and HN, showed higher accumulation of total protein and amino acids in both the leaves and the roots, whereas LA-treated plants exhibited increased levels of these compounds compared to LN plants (Table 1). However, the interaction between growth stage and treatment (S x T) significantly (*P* ≤ 0.05) affected only root biomass (Table 2).

**Table 1:** Effect of different nitrogen forms on growth, photosynthesis, total amino acid and protein content in the leaf and root of maize

Shoot/leaf	20 DAT					40 DAT		
	LN	MN	HN	LA	LN	MN	HN	LA
Biomass DW (g)	0.236 ± 0.02c	0.318 ± 0.04b	0.521 ± 0.02a	0.294 ± 0.03b	0.5651 ± 0.02c	1.1541 ± 0.04b	1.537 ± 0.14a	0.861 ± 0.08c
Root/shoot ratio	0.592 ± 0.06a	0.282 ± 0.05b	0.143 ± 0.01c	0.351 ± 0.04b	0.7972 ± 0.06a	0.2675 ± 0.03c	0.096 ± 0.0s1d	0.375 ± 0.04b
Total biomass DW (g)	0.311 ± 0.02c	0.402 ± 0.04c	0.619 ± 0.02a	0.432 ± 0.04c	0.707 ± 0.03d	1.462 ± 0.05b	1.986 ± 0.16a	1.193 ± 0.08c
Pn [μmol (CO_2_) m^−2^ s^−1^]	20.066 ± 0.53d	27.111 ± 0.99b	32.088 ± 1.22a	23.022 ± 0.95c	22.158 ± 2.31d	31.202 ± 1.24b	35.287 ± 0.80a	27.100 ± 1.10c
Nitrogen (μmol/g DW)	61.166 ± 4.50d	83.189 ± 2.86b	99.238 ± 4.39a	74.530 ± 3.81c	66.968 ± 3.19d	99.758 ± 1.32b	112.075 ± 3.72a	85.825 ± 1.99c
Amino acid (μmol/g FW)	14.730 ± 0.64d	22.935 ± 1.12b	28.767 ± 1.16a	18.633 ± 1.27c	10.800 ± 0.52d	20.969 ± 1.32b	26.532 ± 1.68a	13.557 ± 0.78c
Total protein (μmol/g FW)	29.461 ± 1.28d	47.535 ± 2.78b	57.530 ± 2.31a	37.266 ± 2.58c	21.601 ± 1.05d	41.900 ± 2.64b	53.060 ± 3.35a	27.113 ± 1.56c

Root		20 DAT				40 DAT		
	LN	MN	HN	LA	LN	MN	HN	LA
Biomass DW (g)	0.138 ± 0.004a	0.087 ± 0.02c	0.075 ± 0.01d	0.101 ± 0.004b	0.449 ± 0.03a	0.308 ± 0.01c	0.14 ± 0.02d	0.321 ± 0.02b
Root/shoot ratio	0.592 ± 0.06a	0.282 ± 0.05b	0.143 ± 0.01c	0.351 ± 0.04b	0.797 ± 0.06a	0.2675 ± 0.03c	0.096 ± 0.0s1d	0.375 ± 0.04b
Nitrogen (μmol/g DW)	23.733 ± 0.77d	35.969 ± 1.60b	49.535 ± 3.45a	29.365 ± 2.10c	24.534 ± 0.71d	32.491 ± 0.96b	41.098 ± 1.81a	30.158 ± 0.55c
Amino acid (μmol/g FW)	8.376 ± 0.75d	15.345 ± 1.79b	20.568 ± 0.64a	12.310 ± 0.81c	6.545 ± 0.72d	9.323 ± 0.52b	11.330 ± 0.75a	8.553 ± 0.64c
Total protein (μmol/g FW)	16.739 ± 1.50d	30.657 ± 3.59b	41.889 ± 1.60a	24.630 ± 1.62c	13.060 ± 1.44d	18.467 ± 1.03b	22.669 ± 1.51a	17.067 ± 1.28c

Data are presented as mean ± SE (*n* = 6). Statistical significance was determined using Tukey’s multiple range test (*P* ≤ 0.05), with different letters indicating significant differences between treatments. *FW* fresh weight; *LN* low nitrogen (N deficiency); *MN* moderate nitrogen; *HN* high nitrogen; *LA* low ammonium treatment

**Table 2:** Two-way analysis of variance (ANOVA) for the indicators studied in the shoot/leaf and root of maize inbred line TX-40 J under different N treatments

Trait/(plant)	Sources of variation					
	Shoot/leaf			Root		
	Growth stage (S)	Treatment (T)	S x T	Growth stage (S)	Treatment (T)	S x T
	(df = 1)	(df = 3)	(df = 3)	(df = 1)	(df = 3)	(df = 3)
Biomass	0.0046**	0.0017**	0.5867 ns	0.0079**	0.0053**	0.0168*
Total biomass	0.0024**	0.0469*	0.1272 ns	0.4841 ns	0.0069**	0.0933 ns
Pn	0.0828 ns	0.0057**	0.6957 ns	n/a	n/a	n/a
Sucrose	0.3649 ns	0.00478**	0.6724 ns	0.001**	0.0065**	0.0761 ns
Nitrogen	0.0066**	0.0146*	0.5013 ns	0.0724 ns	0.0022**	0.2929 ns
Soluble sugar	0.0084**	0.0031**	0.1439 ns	0.0029**	0.0081**	0.0093**
Sucrose	0.3649 ns	0.0047**	0.6724 ns	0.001**	0.0065**	0.0761 ns
Starch	0.0021**	0.0032**	0.0677 ns	0.004**	0.0047**	0.3294 ns
Fructose	0.0522 ns	0.0125*	0.1901 ns	0.0381*	0.0086**	0.5235 ns
Glucose	0.5173 ns	0.0044**	0.1753 ns	0.2566 ns	0.0158*	0.3824 ns
Hexose	0.1592 ns	0.0008***	0.1495 ns	0.1522 ns	0.0007***	0.1695 ns
TNC	0.002**	0.0005***	0.1207 ns	0.0065**	0.0023**	0.1237 ns
H/S ratio	0.3255 ns	0.0101*	0.4445 ns	0.0151*	0.1462 ns	0.0469*
SPS	0.0084**	0.0031**	0.1439 ns	0.0147*	0.0062**	0.0315*
SuSy	0.0355*	0.0087**	0.7709 ns	0.0032**	0.0152*	0.5101 ns
AGPase	0.0064**	0.0563 ns	0.2363 ns	0.438 ns	0.0063**	0.2436 ns
CINV	0.1131 ns	0.003**	0.7025 ns	0.1801 ns	0.0073**	0.0445*
VINV	0.0438*	0.0115*	0.1266 ns	0.019*	0.0003***	0.0605 ns
CWINV	0.0005***	0.0162*	0.1512 ns	0.0011**	0.0057**	0.4607 ns
Amino acid	0.0209*	0.0005***	0.6194NS	0.0199*	0.0131*	0.1075 ns
Total protein	0.031*	0.0023**	0.7153 ns	0.0236*	0.0092**	0.0999 ns
TSUC	0.0201*	0.0036**	0.5331 ns	0.0078**	0.003**	0.0184*
*ZmSuSy1*	0.2054 ns	0.0195*	0.3628 ns	0.1572 ns	0.0154*	0.8374 ns
*ZmSPS1*	0.6194 ns	0.0183*	0.4934 ns	0.4353 ns	0.0384*	0.8592 ns
*ZmCINV1*	0.0387*	0.0169*	0.645 ns	0.673 ns	0.0011**	0.8434 ns
*ZmVINV1*	0.6256 ns	0.0097**	0.6616 ns	0.1943 ns	0.0482*	0.1635 ns
*ZmCWINV1*	0.0428*	0.009**	0.8617 ns	0.7135 ns	0.0074**	0.2518 ns
*ZmAGPase1*	0.0215*	0.0121*	0.3896 ns	0.3421 ns	0.0407*	0.1725 ns
*ZmSUC2*	0.0602 ns	0.0026**	0.7507 ns	0.0049**	0.0169*	0.6381 ns
*ZmSWEET14*	0.9827 ns	0.0582 ns	0.9378 ns	0.0119*	0.0225*	0.3619 ns
*ZmSS1*	0.0428*	0.009**	0.8617 ns	0.7137 ns	0.0074**	0.2518 ns
*ZmAMY1*	0.04644*	0.0183*	0.4934 ns	0.6441 ns	0.0016**	0.8302 ns
*ZmBAM1*	0.0253*	0.0074**	0.4297 ns	0.1206 ns	0.0370*	0.1709 ns
SS	0.0004***	< 0.0001****	0.0632 ns	0.01801**	0.0017**	0.1221 ns
AMY	0.0047**	< 0.0001***	0.0413*	0.011*	< 0.0001****	0.0919 ns
BAM	0.0449*	0.001**	0.0061**	0.0277*	0.0021**	0.5368 ns

*R/S ratio* root:shoot ratio; *Pn* net photosynthetic rate; *H/S ratio* hexose/sucrose ratio; *SuSy* sucrose synthase activity; CINV/*ZmCINV1*, cytoplasmic invertase activity; VINV/*ZmVINV1*, vacuolar invertase activity; SPS/*ZmSPS1*, sucrose phosphate synthase; AGPase/*ZmAGPase1*, ADP-glucose pyrophosphorylase; TSUC, total sucrolytic activity; *TNC* total non-structural carbohydrate; ns, non-significant; *na* non-applicable. In addition, *, **, and *** denote significance at probability levels of 0.05, 0.01, and 0.001, respectively. Tissue biomasses were quantified as g DW, net photosynthesis was quantified as [μmol (CO_2_) m^−2^ s^−1^], while other indicators were quantified as (μmol/g DW)

### Soluble sugars and starch accumulation under different N forms

**Fig. 1:**
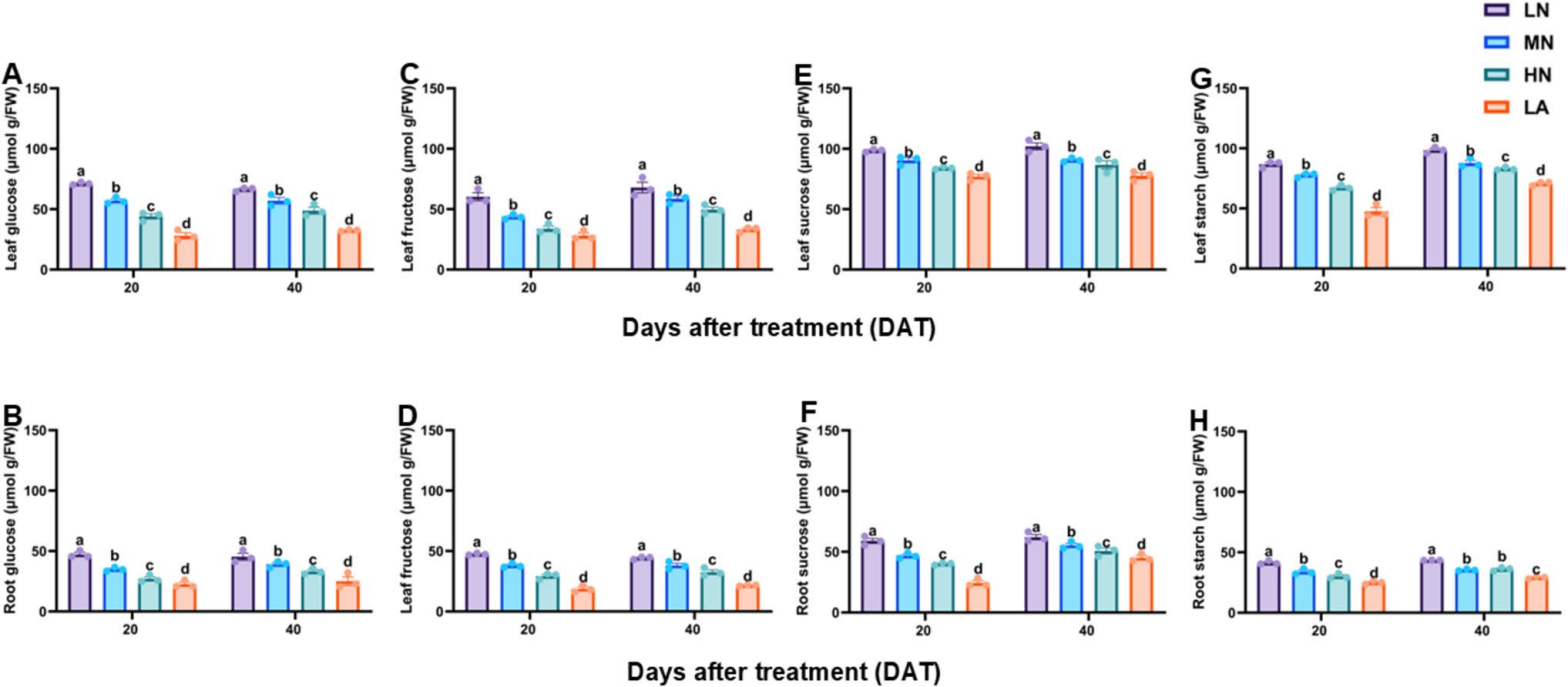
Effect of different nitrogen forms on glucose, fructose, sucrose, and starch content in the leaves (**A**, **C**, **E**, **G**) and roots (**B**, **D**, **F**, **H**) of maize inbred line TX-40 J. Data are presented as mean ± SE (*n* = 6). Statistical significance was determined using Tukey’s multiple range test (*P* ≤ 0.05), with different letters indicating significant differences between treatments. *FW* fresh weight; *LN* low nitrogen (N deficiency); *MN* moderate nitrogen; *HN* high nitrogen; *LA* low ammonium treatment

The glucose, fructose, sucrose, starch, and soluble sugar contents in the leaves (Figs. 1A, C, E, G and S1A) and roots (Figs. 1B, D, F, Hand S1B) were significantly (*P* ≤ 0.05) higher in LN-treated plants. This resulted in a significantly (*P* ≤ 0.05) increased hexose content (sum of glucose and fructose), total non-structural carbohydrate (TNC) content (sum of sucrose, glucose, fructose and starch) and hexose: sucrose (H/S) ratio in both the leaves (Fig. S1C, E and G) and roots (Fig. S1D, F and H). Growth stage S affected leaf soluble sugar, starch, and TNC, as well as root soluble sugar, sucrose, fructose, starch, TNC, and H/S ratio. T affected the leaf and root soluble sugar, sucrose, fructose, glucose, hexose, TNC, and H/S ratios. In contrast, the S x T impacted only the root soluble sugar and H/S ratio (Table 2).

### Sugar and starch metabolism enzymes activity

**Fig. 2:**
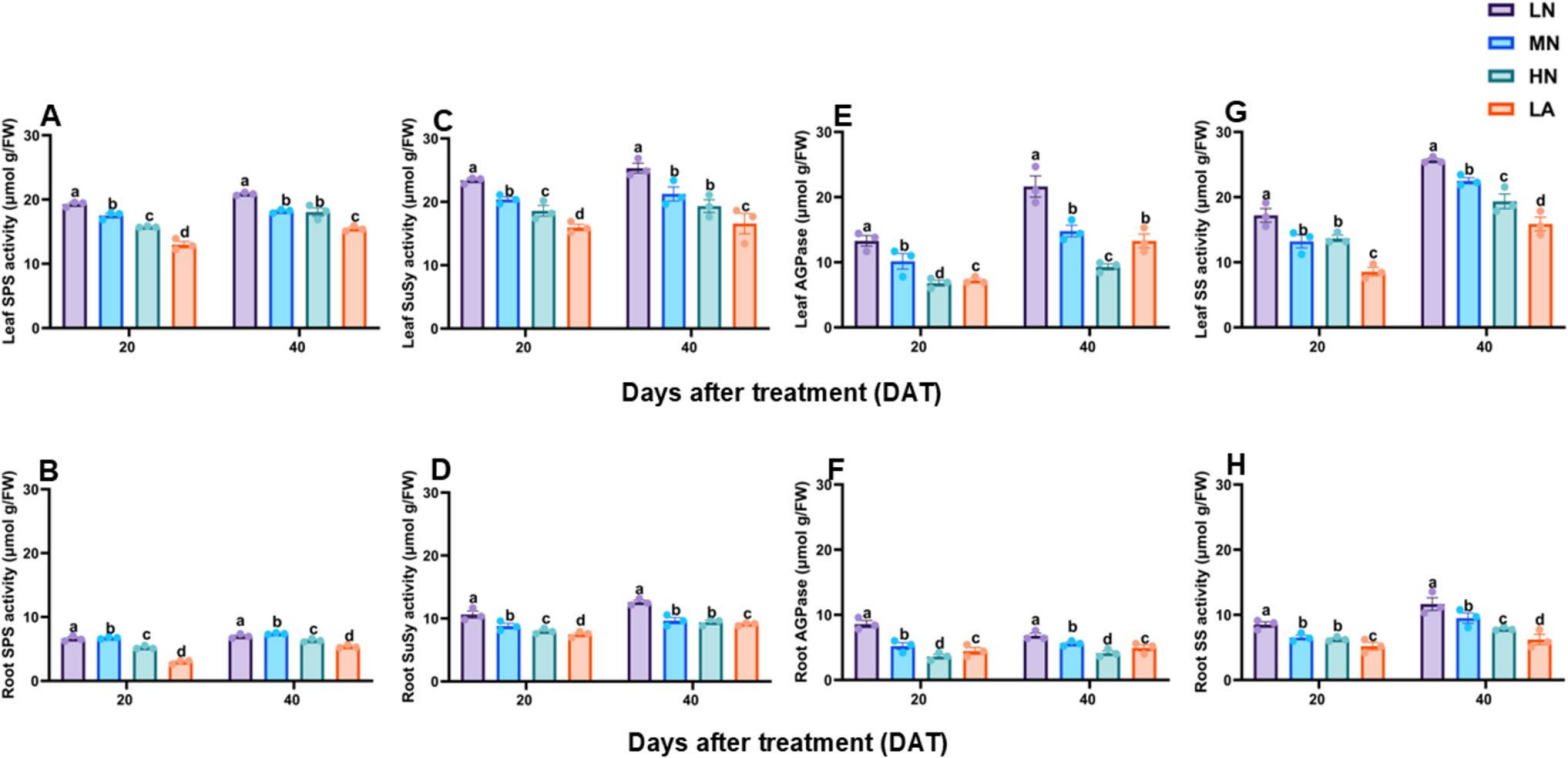
Effect of different nitrogen forms on sucrose phosphate synthase, sucrose synthase, sucrose synthase, ADP-glucose pyrophosphorylase and starch synthase activity in the leaves (**A**, **C**, **E**, **G**) and roots (**B**, **D**, **F**, **H**) of maize inbred line TX-40 J. Data are presented as mean ± SE (*n* = 6). Statistical significance was determined using Tukey’s multiple range test (*P* ≤ 0.05), with different letters indicating significant differences between treatments. *FW* fresh weight; *LN* low nitrogen (N deficiency); *MN* moderate nitrogen; *HN* high nitrogen; *LA* low ammonium treatment

SPS and SuSy activities were significantly (*P* ≤ 0.05) higher in the leaves (Fig. 2A, C) and roots (Fig. 2B, D) of LN-treated plants. Similarly, CINV, VINV, and CWINV activities increased significantly (*P* ≤ 0.05) in the leaves (Fig. S2A, C, E) and roots (Fig. S2B, D, F) of LN-treated plants, resulting in higher total sucrolytic activity in the leaves (Fig. S2G) and roots (Fig. S2H) in LN plants compared to plants in other treatment groups. In addition, plants under LN treatment showed enhanced AGPase, SS, AMY, and BAM activity in the leaves (Figs. 2E, G and S3A, C) and roots (Figs. 2E, G and S3B, D), which supported the higher starch accumulation in LN-nourished plants. Both, growth stage (S) and N treatment (T), significantly (*P* ≤ 0.05) influenced leaf and root SPS, SuSy, AGPase, SS, AMY, BAM, CINV, VINV, CWINV, and total sucrolytic activity, while S x T interaction was found for leaf AMY and BAM activities, as well as root SPS, SuSy, AGPase, and total sucrolytic activity (Table 2).

### Expression pattern of sugar and starch metabolism-related gene activity

**Fig. 3:**
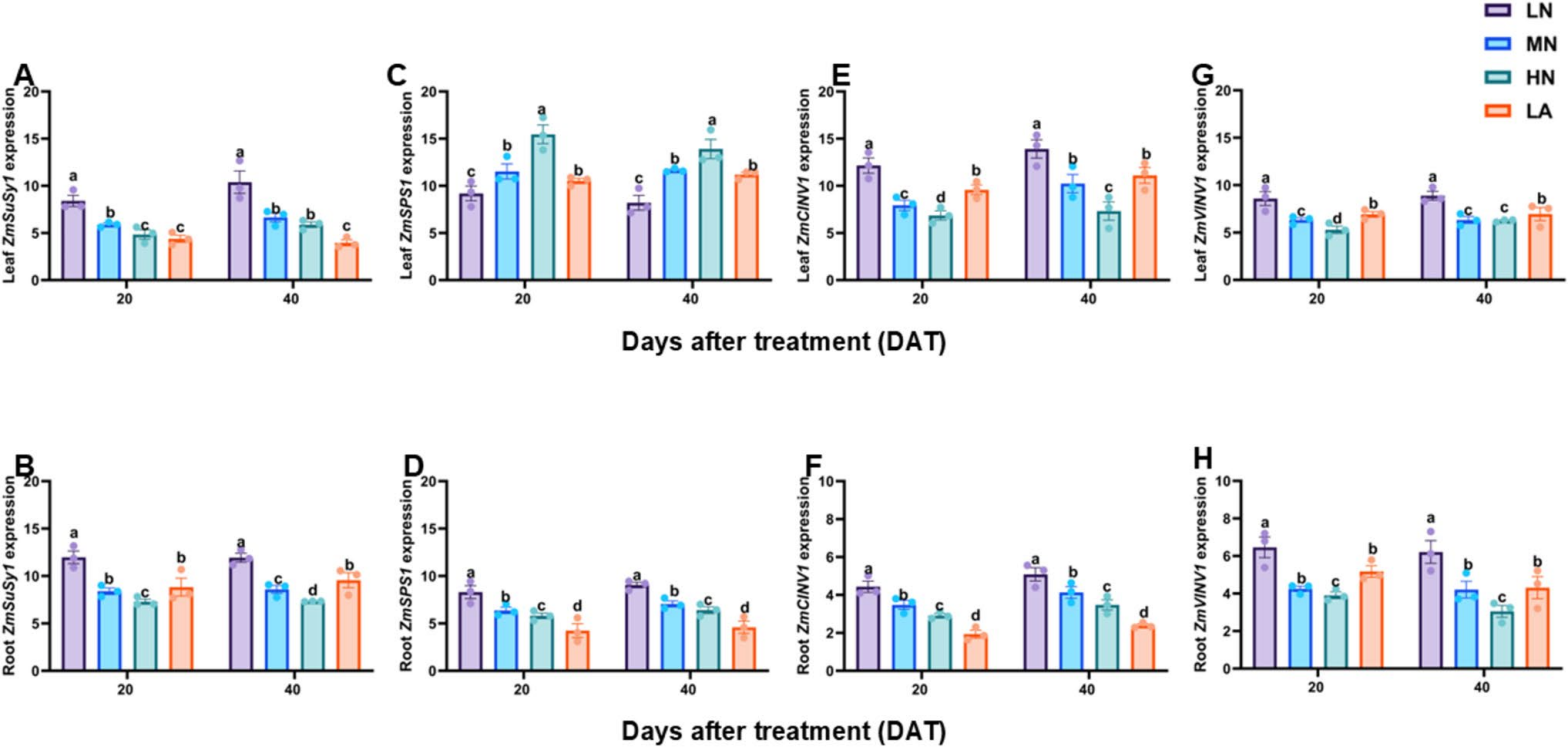
Effect of different nitrogen forms on the expression pattern of sugar-metabolizing genes. Expression level of *ZmSuSy1*, *ZmSPS1*, *ZmCINV1*, and *ZmVINV1* in the leaves (**A**, **C**, **E**, **G**) and roots (**B**, **D**, **F**, **H**) of maize inbred line TX-40 J. Data are presented as mean ± SE (*n* = 6). Statistical significance was determined using Tukey’s multiple range test (*P* ≤ 0.05), with different letters indicating significant differences between treatments. *FW* fresh weight; *LN* low nitrogen (N deficiency); *MN* moderate nitrogen; *HN* high nitrogen; *LA* low ammonium treatment

**Fig. 4:**
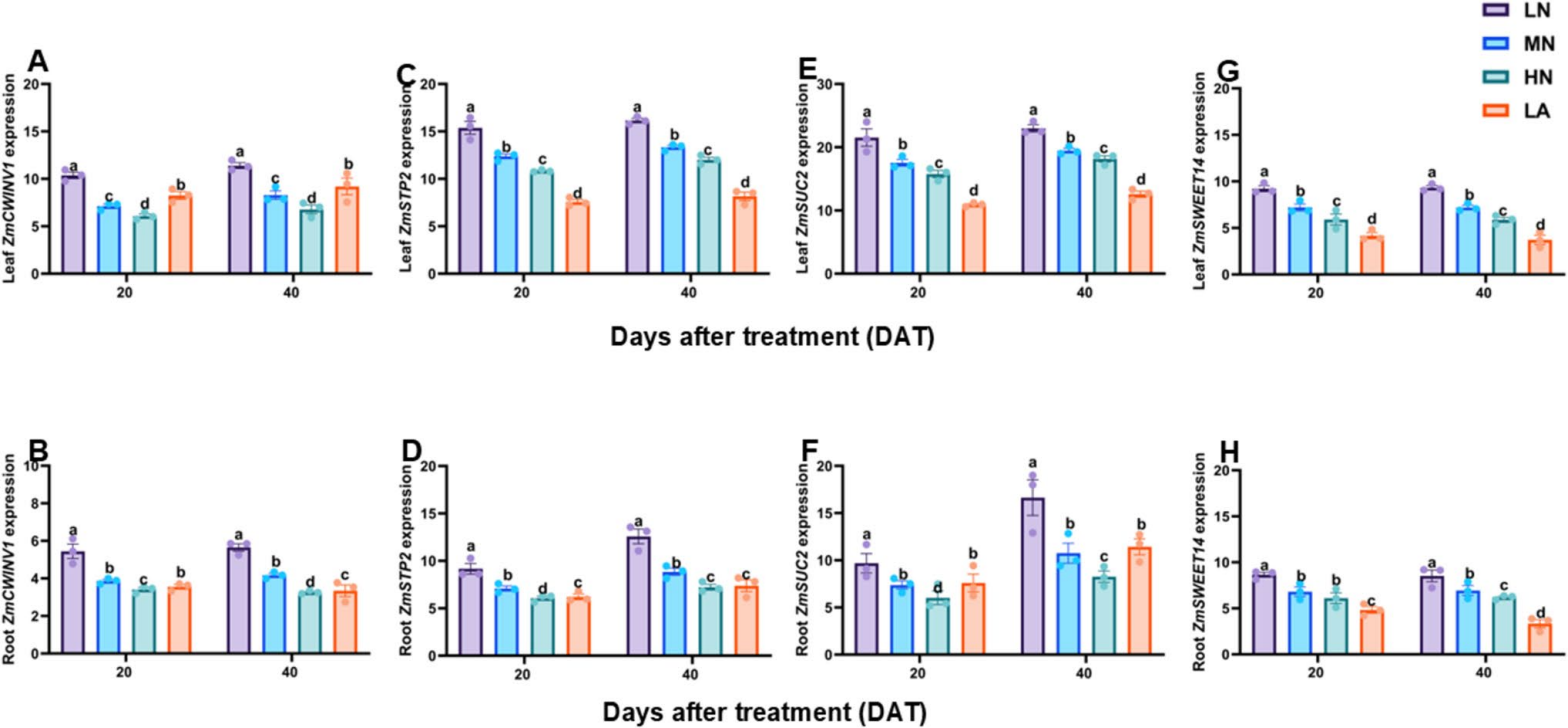
Effect of different nitrogen forms on the expression pattern of sugar-metabolizing and sucrose transporter genes. Expression level of *ZmCWINV1*, *ZmSTP2*, *ZmSUC2*, and *ZmSWEET14* in the leaves (**A**, **C**, **E**, **G**) and roots (**B**, **D**, **F**, **H**) of maize inbred line TX-40 J. Data are presented as mean ± SE (*n* = 6). Statistical significance was determined using Tukey’s multiple range test (*P* ≤ 0.05), with different letters indicating significant differences between treatments. *FW* fresh weight; *LN* low nitrogen (N deficiency); *MN* moderate nitrogen; *HN* high nitrogen; *LA* low ammonium treatment

**Fig. 5:**
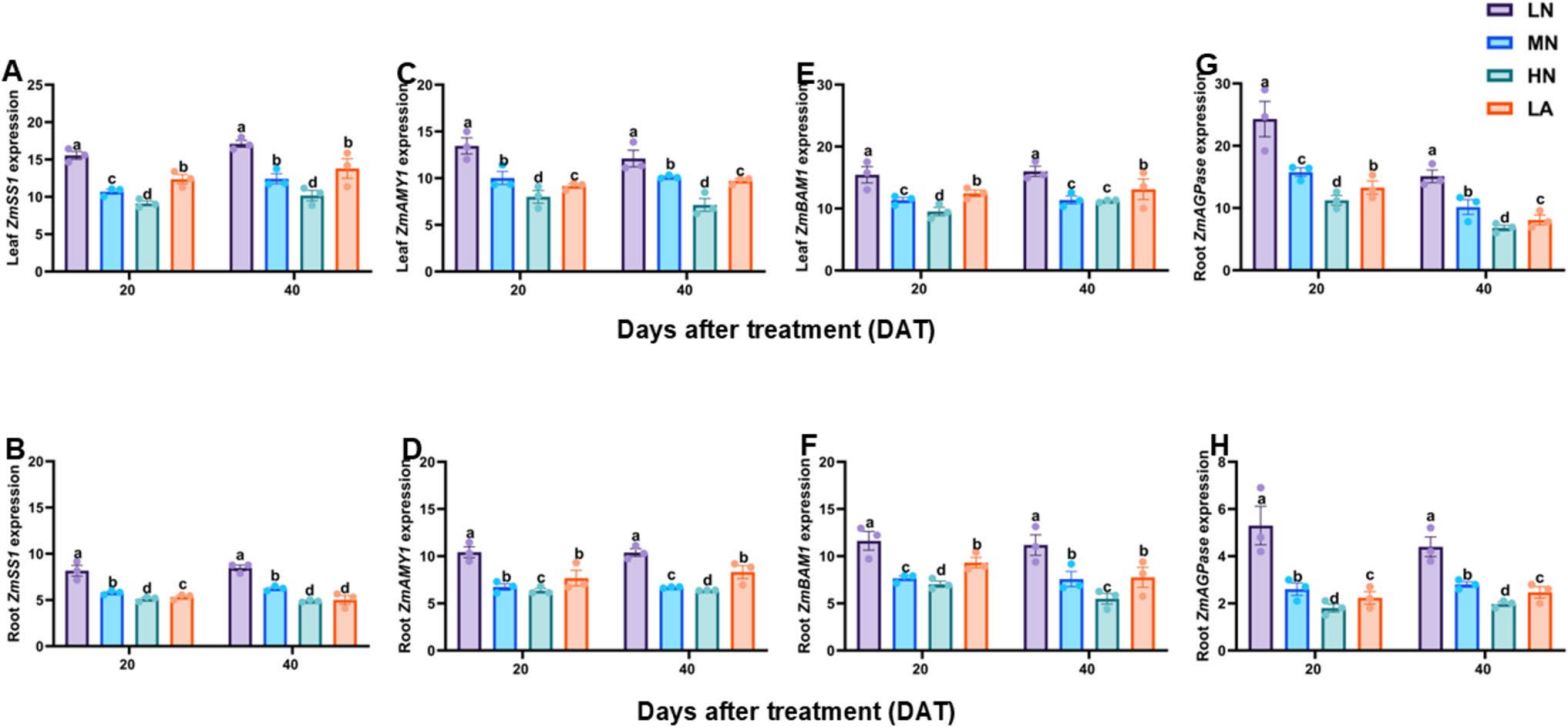
Effect of different nitrogen forms on the expression pattern of starch metabolizing genes. Expression level of *ZmSS1*, *ZmAMY1*, *ZmBAM1*, and *ZmAGPase1* in the leaves (**A**, **C**, **E**, **G**) and roots (**B**, **D**, **F**, **H**) of maize inbred line TX-40 J. Data are presented as mean ± SE (*n* = 6). Statistical significance was determined using Tukey’s multiple range test (*P* ≤ 0.05), with different letters indicating significant differences between treatments. *FW* fresh weight; *LN* low nitrogen (N deficiency); *MN* moderate nitrogen; *HN* high nitrogen; *LA* low ammonium treatment

To understand the molecular mechanisms associated with C accumulation under different N forms, the expression levels of sucrose and starch metabolism-related genes *ZmSuSy1, ZmSPS1*, *ZmCINV1*, *ZmVINV1, ZmCWINV1*, *ZmSTP2*, *ZmSUC2*, *ZmSWEET14*, *ZmSS1*, *ZmAMY1*, *ZmBAM1*, and *ZmAGPase1* were analyzed in the leaves and roots of maize plants under LN, MN, HN, and LA treatments. As shown in Figs. 3, 4, and 5, the sugar and starch metabolism-related genes were differentially regulated in the leaves and roots of maize plants. For example, *ZmSuSy1*, *ZmSPS1*, *ZmCINV1*, *ZmVINV1*, *ZmSTP2*, *ZmSUC2*, and *ZmSWEET14* were highly upregulated in the leaves (Figs. 3, 4A, C, E) and roots (Figs. 3, 4B, D, F, H). In contrast, the starch metabolism-related genes *ZmSS1*, *ZmAMY1* and *ZmBAM1* and *ZmAGPase1* were highly upregulated in both the leaves (Fig. 5A, C, E, G) and roots (Fig. 5B, D, F, H) of maize plant under LN treatment. In addition, growth stage (S) greatly influenced the expression of *ZmCWINV1*, *ZmCINV1*, and *ZmSTP2*, *ZmSS, ZmAMY1* and *ZmBAM1* in the leaves and *ZmSUC2* and *ZmSWEET14* in the roots. Nitrogen treatment (T) affected the expression of all genes in the leaves and roots, except for *ZmSWEET14* in the leaves. However, S x T had no influence on the expression of any genes in both the leaves and roots of maize plants (Table 2).

### Diurnal changes of sugars and starch under different N forms

**Fig. 6:**
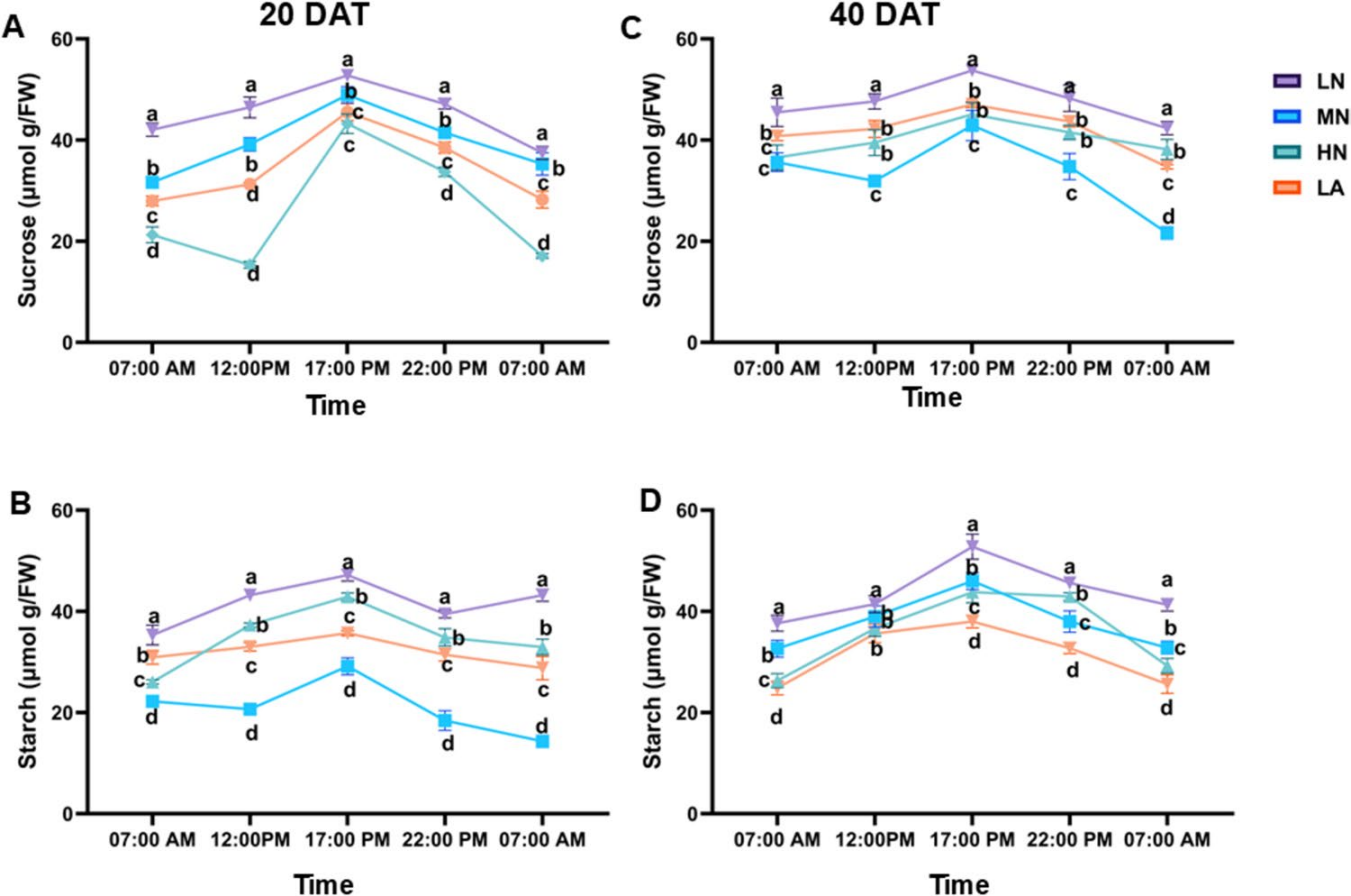
Diurnal changes in leaf sucrose (**A**) and starch (**B**) at 20 days after treatment (DAT) and leaf sucrose (**C**) and starch (**D**) at 40 DAT under different nitrogen treatments. Samples were collected at 7:00, 12:00, 17:00, 22:00, and 7:00 on the second day. Data are presented as mean ± SE (*n* = 6). Statistical significance was determined using Tukey’s multiple range test (*P* ≤ 0.05), with different letters indicating significant differences between treatments. *FW* fresh weight; *LN* low nitrogen (N deficiency); *MN* moderate nitrogen; *HN* high nitrogen; *LA* low ammonium treatment

The diurnal pattern of sucrose, starch, fructose, and glucose levels showed the lowest values in the early morning (7:00 AM), followed by an increase at 12:00 PM, peaking at 5:00 PM, and declining overnight to reach their lowest levels again at 7:00 AM the next day. This pattern was consistent across both 20 DAT and 40 DAT (Figs. 6A–D and S4A-D). At 20 DAT, significant differences (*P* ≤ 0.05) in sucrose and starch contents were recorded among the treatment groups. Plants subjected to LN treatment consistently exhibited elevated concentrations of leaf sucrose, starch, fructose, and glucose throughout the day. These sugar levels were significantly different (*P* ≤ 0.05) from those observed in other treatment groups during both sampling stages (Figs. 6A–D and S4A–D). Furthermore, after overnight transport, plants under LN treatment retained significantly (*P* ≤ 0.05) higher leaf sucrose and starch concentrations than those in other treatment groups (Fig. 6A–D).

### Leaf sucrose and starch synthesis and degradation under different nitrogen forms

**Fig. 7:**
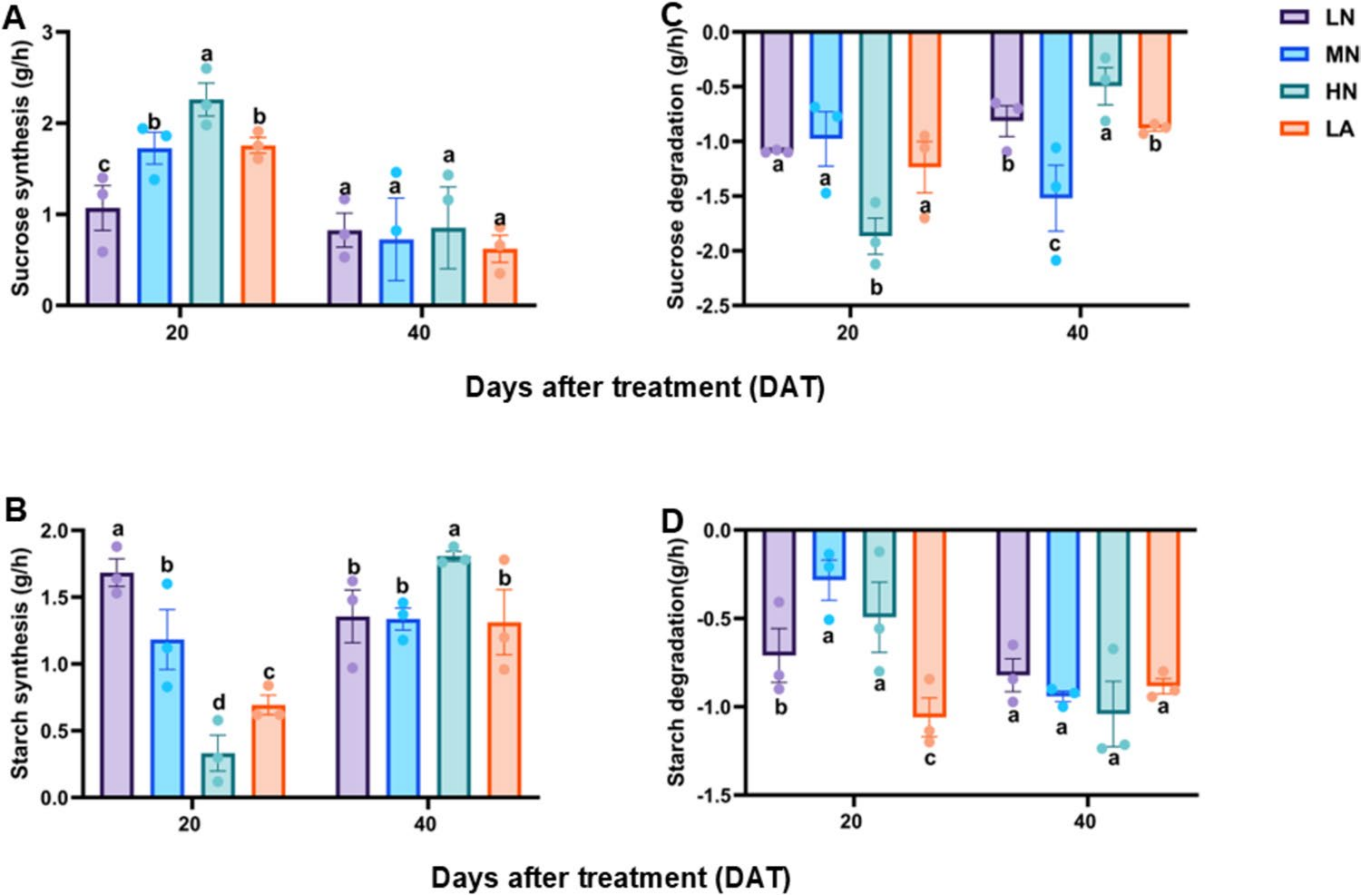
Sucrose (**A**) and starch (**B**) synthesis and sucrose (**C**) and starch (**D**) degradation in the leaves of maize inbred line TX-40 J grown under varying nitrogen treatments. Data are presented as mean ± SE (*n* = 6). Statistical significance was determined using Tukey’s multiple range test (*P* ≤ 0.05), with different letters indicating significant differences between treatments. FW, fresh weight; LN, low nitrogen (N deficiency); *MN* moderate nitrogen; *HN* high nitrogen; *LA* low ammonium treatment

Starch synthesis significantly increased in the leaves of LN-treated plants at 20 DAT (Fig. 7). However, MN-treated plants exhibited higher leaf sucrose and starch synthesis rates, which were comparable to those observed in LN-, HN-, and LA-treated plants (Fig. 7A, B). Furthermore, sucrose and starch degradation rates were consistently higher at both 20 and 40 DAT in plants subjected to LN treatment compared to other treatments (Fig. 7C, D). Plants under MN treatment showed significantly higher net sucrose accumulation. Meanwhile, net starch accumulation was comparable between LN- and MN-treated plants at 20 DAT, and between LN- and HN-treated plants at 40 DAT (Fig. S5A–B).

### Diurnal and spatial distribution of soluble sugars and starch under different forms

**Fig. 8:**
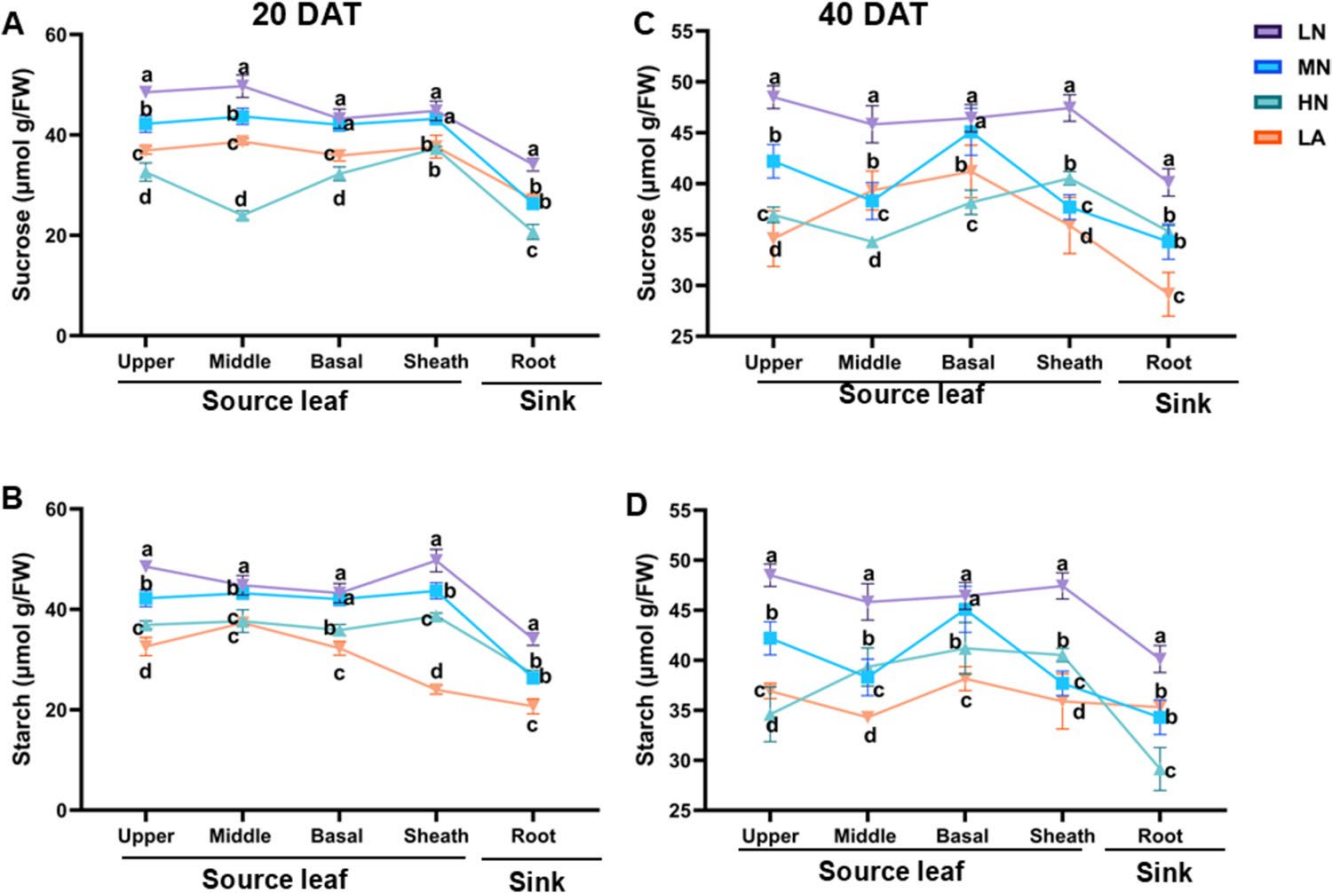
Leaf sucrose (**A**) and starch (**B**) at 20 DAT, and leaf sucrose (**C**) and starch (**D**) at 40 DAT in different tissues under various nitrogen form treatments. Data are presented as mean ± SE (*n* = 6). Statistical significance was determined using Tukey’s multiple range test (*P* ≤ 0.05), with different letters indicating significant differences between treatments. *FW* fresh weight; *LN* low nitrogen (N deficiency); *MN* moderate nitrogen; *HN* high nitrogen; *LA* low ammonium treatment

Plants subjected to LN treatment exhibited significantly higher sucrose and starch contents across various maize tissues (upper, middle, and basal leaves, the leaf sheath, and the roots) compared to other treatment groups. Specifically, at 21:00, the upper, middle, and basal leaves, as well as the leaf sheath, demonstrated elevated sucrose levels. In contrast, plants under HN treatment displayed reduced sucrose accumulation by the end of the day (Fig. 8A). By 07:00 the following morning (end of the night), LN-treated plants retained higher residual sucrose levels in the upper and basal leaves and the leaf sheath, with concentrations comparable to those recorded at 21:00 (Fig. 8A, C). Similarly, LN-treated plants accumulated greater starch levels in the leaves by 21:00 and retained higher starch concentrations following overnight remobilization. Starch content in the roots (sink tissues), however, was relatively lower and showed significant variation among treatment groups at both 21:00 and 07:00 (Fig. 8B–D).

As shown in Fig. S6, LN-treated plants exhibited higher glucose and fructose levels across all examined maize tissues. The upper, middle, basal, and sheath regions, which constitute the leaf tissues, accumulated significantly higher fructose levels, particularly at 21:00 compared to 07:00. In contrast, LA-treated plants displayed lower glucose and fructose concentrations at 21:00 (Fig. S6A–D). By 07:00, LN-treated plants retained greater residual glucose and fructose in the leaves, mirroring the concentrations observed at 21:00. Interestingly, fructose and glucose levels were consistently lower in root tissues (sink tissues) compared to leaves (source tissues), with notable variation among treatment groups at both sampling times (Fig. S6A–D). This pattern underscores the influence of N availability on carbohydrate storage and redistribution. LN treatment notably enhanced the retention of sucrose, starch, glucose, and fructose in source tissues, even after overnight remobilization.

### Correlations of R/S ratio with physio-biochemical and molecular indicators

To investigate the relationship between the R/S ratio and various indicators in maize leaves and roots under different nitrogen (N) conditions, a Pearson correlation analysis was conducted (Table 3). In maize leaves, the R/S ratio exhibited significant positive or negative correlations with the majority of the indicators, with the exception of H/S ratio and TNC. Similarly, in maize roots, the R/S ratio significantly correlated (positively or negatively) with most indicators. However, no significant correlations were observed with net photosynthetic rate Pn, total protein, glucose, fructose, hexose, H/S ratio, TNC, or the expression levels of *ZmCINV1* and *ZmSWEET14* (Table 3).

**Table 3:** Pearson’s correlation analysis of R/S ratio with physio-biochemical and molecular indicators in the leaves and root of maize. *, **, ***, ns denote P < 0.05, 0.01, 0.001, and not significant, respectively

Traits/(plants)	Leaf	Root
Shoot biomass	−0.941**	0.968***
Total biomass	−0.921**	−0.921**
Nitrogen	−0.978**	−0.921**
Net photosynthetic rate	−0.957**	−0.924**
Soluble sugar	0.548**	0.235**
Sucrose	0.641**	0.574**
Total amino acid content	−0.926**	−0.967**
Total protein content	−0.926**	−0.959**
Starch content	0.788**	0.948**
Fructose content	0.638**	0.544**
Glucose content	0.544**	0.678**
Hexose content	0.583**	0.583**
Hexose/sucrose ratio	0.482**	0.657**
Total non-structured carbohydrates	0.341 ns	0.404**
Sucrose phosphate synthase	0.548**	0.235**
Sucrose synthase	0.658**	0.850**
Cytoplasmic invertase	0.876**	0.896**
Vacuolar invertase	0.999**	0.980**
Cell wall invertase	0.981**	0.963**
Total sucrolytic activity	0.937**	0.963**
AGPase activity	0.935**	0.947**
Starch synthase	0.807**	0.871**
α-amylase	0.856**	0.998**
β-amylase	0.871**	0.958**
*ZmSuSy1*	0.772**	0.624**
*ZmSPS1*	−0.948**	0.986**
*ZmCINV1*	0.990**	0.585**
*ZmVINV1*	0.996**	1.000**
*ZmCWNV1*	0.995**	0.908*
*ZmSTP2*	0.996**	1.000**
*ZmSUC2*	0.511**	0.997**
*ZmSWEET14*	0.617**	0.955**
ZmAGPase1	0.934**	0.547**
*ZmSS1*	0.995**	0.576**
*ZmAMY1*	0.959**	0.558**
*ZmBAM1*	0.994**	0.640**

## Discussion

### LN inhibits shoot development and N assimilation while promoting root biomass allocation

Maize depends greatly on N for growth and development, and studies have shown that different N forms distinctly influence plant physiological responses (Nematpour and Eshghizadeh 2024). In this study, LN treatment significantly (*P* ≤ 0.05) reduced total biomass due to suppressed photosynthesis and C fixation. Despite overall biomass reduction under LN, the enhanced root growth resulted in a higher R/S ratio (Table 1). This shift reflects an adaptive strategy to allocate limited C toward root systems to improve nutrient acquisition under N deficiency (Zhao et al. 2020). Moreover, plants grown under MN and HN treatments accumulated more amino acids, proteins, and total N, correlating with enhanced growth (Table 1). These findings highlight the essential role of adequate NO₃⁻ supply in promoting N assimilation and physiological performance (Wang et al. 2021b). In contrast, LN impaired amino acid and protein synthesis, further limiting shoot development. Our findings underscore the strong interaction between N and C metabolism, as reduced N availability disrupted photosynthesis and led to altered biomass partitioning (Wang et al. 2021a). The findings emphasize the role of LN in maintaining the balance between N and C metabolism, which is essential for maximizing plant growth, nitrogen use efficiency (NUE), and overall productivity under N-deficiency condition (Aluko et al. 2023).

### LN increased sugars and starch accumulation in leaves, optimizing C allocation and sustaining growth under N deficiency

Efficient coordination between C fixation and downstream metabolism is essential for plant growth and development. LN inhibited photosynthetic C assimilation, resulting in metabolic disruption that constrained growth (Table 1). However, maize leaves under LN accumulated higher sugars and non-structured carbohydrates (Figs. 1A, C, E and S1A, C, E, G). The observed sugar accumulation reflects and adaptive shift in C metabolism, supported by enhanced activities and upregulation of sucrose metabolism enzymes, SPS, SuSy, CINV, VINV, and CWIN and their associated genes, *ZmSPS1*, *ZmSuSy1*, *ZmCINV1*, *ZmVINV1* and *ZmCWINV1* (Figs. 2A, C. E, G and 4 A). Although sucrolytic enzymes activity and their corresponding genes were upregulated under LN, sucrose level remained higher in the leaves, which may be due to enhanced biosynthesis and turnover, as indicated by elevated SPS and SuSy activities and simultaneous synthesis and degradation (Fig. 2A, B, C, D) supporting energy demands while maintaining sucrose for osmotic and signaling functions (Bilska-Kos et al. 2020). Furthermore, a positive correlation was observed between R/S ratio and the level of sugars in LN plants (Table 3), indicating a preferential partitioning of C to the root systems as a strategic adaptation to N limitation. LN upregulated the expression of sucrose transporter genes, *ZmSUC2*, *ZmSTP2* and *ZmSWEET14* in both the leaves (Fig. 4C, E, G) and roots (Fig. 4D, F, H) of maize seedlings, indicating enhanced source-to-sink movement, improved phloem loading and unloading efficiency, and C partitioning to the roots to support growth during N stress (Lemoine et al. 2013; Saddhe et al. 2021; Li et al. 2024). Furthermore, the significantly increased root sugar and starch metabolism, along with increased root ratios in maize seedlings under LN can be attributed to the CO₂-concentrating mechanism in C₄ plants, which enables reduced abundance of ribulose-1,5-bisphosphate carboxylase/oxygenase (Rubisco) and enhances NUE (Evans and Clarke 2019). Unlike C₄ species such as maize, C₃ plants like wheat depend greatly on Rubisco to maintain efficient photosynthesis, requiring greater N investment in the photosynthetic apparatus. In contrast, maize can strategically allocate more N toward root development and stress adaptation (Lemaire et al. 2008). This suggests that the reduced leaf N content observed in LN-treated maize likely facilitated increased N investment in root biomass and sink strength. C₃ plants, by comparison, may experience greater constraints under N limitation due to the higher N demands associated with maintaining photosynthetic capacity (Gao et al. 2015; Mu and Chen 2021). Future research will explore the mechanistic links between N investment in Rubisco and belowground resource allocation under varying N regimes across photosynthetic types. Comparative analyses between C₃ and C₄ species will provide a comprehensive insight into N budgeting strategies and their implications for root development, stress adaptation, and overall nitrogen use efficiency.

Starch metabolism responds dynamically to N availability, reflecting its role in adaptive C storage under N-limited conditions (Zhao et al. 2020). LN induced starch accumulation (Fig. 1G–H), with upregulation of starch biosynthetic (*ZmSS1*, *ZmAGPase1*,) (Fig. 5A–B, G–H) and degradative (*ZmAMY1*, *ZmBAM1*) (Fig. 4C–D, E–F) genes and their corresponding enzymes (Figs. 2E–H, S3A–D). These dynamic responses reflect a tightly regulated starch turnover system that adjusts to N deficiency by balancing energy storage and mobilization (Smith and Zeeman 2020). A positive correlation was observed between R/S ratio, starch levels, metabolizing enzymes activity, and their associated genes (Table 3), which reinforces the central role of starch metabolism in the adaptive response of maize to N limitation (Kumar et al. 2023).

### LN stimulates root carbohydrate metabolism for adaptive growth

Sugar metabolism is crucial for root development under N deficiency (Zhao et al. 2020). LN increase root SPS, SuSy and INVs activities (Figs. 2 and S2B, D, F, H), along with upregulation of *ZmSPS1*, *ZmSuSy1*, *ZmCINV1*, *ZmVINV1*, and *ZmCWINV1* (Figs. 3B, D, F and H and 4B), promoting sucrose breakdown and re-synthesis cycle. This led to an increased root sucrose content, correlating with increased enzyme activity and gene expression. Furthermore, starch metabolism was enhanced under LN, evidenced by increased SS, AGPase, AMY, and BAM activities (Figs. 2F, H and S3B, D), and expression of *ZmSS1*, *ZmAGPase1*, *ZmAMY1*, and *ZmBAM1* (Fig. 5B, D, F and H). These dynamic changes increased root starch and sugar content (Fig. 1), supporting stress resilience and improved root growth, as highlighted by Du et al. (2020b). Increased root sink strength likely enhanced sucrose export from the leaves, supporting shoot-to-root C allocation. Interestingly, the R/S ratio correlated positively with sugar content, sugar-metabolizing enzyme activities, and the expression of related genes (Table 3), emphasizing that the intensified recycling and storage of sucrose and starch contributed to an increased R/S ratio and improved adaptation to N limitation.

### Diurnal regulation of carbohydrate metabolism by N form and availability

Carbohydrate levels followed diel rhythms, with LN plants exhibiting consistently higher sugar and starch levels across the photoperiods (Fig. 6A–D). Peak accumulation of sucrose and starch occurred by 17:00, followed by overnight declines, corresponding with photosynthetic accumulation and nighttime utilization via respiration (Amoah and Kaiser 2025). LN plants maintained elevated sucrose and starch levels even after nocturnal export, indicating robust storage and mobilization. Starch synthesis peaked under LN (Fig. 7B), demonstrating an adaptive mechanism for C storage when growth is limited by N (Zhao et al. 2020). Interestingly, MN-treated plants showed the highest overall sucrose and starch synthesis rates (Fig. 7A, B), reflecting a more balanced C/N status. Furthermore, starch and sucrose degradation was enhanced under LN (Fig. 8C, D), promoting rapid energy mobilization (Ma et al. 2024). Although MN increases sucrose accumulation, LN and HN plants showed comparable net starch accumulation at 40 DAT (Fig. S5A–B), revealing distinct metabolic priorities under different N regimes. LN promoted a dynamic turnover, while MN emphasized storage. These patterns highlight the importance of considering both N form and availability in regulating C metabolism.

### N form and availability modulate tissue-specific carbohydrate distribution

Maize plants exhibited differential response to N availability, with LN showing the sugar and starch accumulation (Figs. 8A–D and S6A–D), particularly in the upper leaves at both 20 and 40 DAT. These plants also showed lower N content and distinct diurnal and spatial carbohydrate dynamics (Figs. 8 and S6 and Table 1). While significant variations in C accumulation in the leaves of plants under different N forms have been documented in some species (Lv et al. 2021), the present study confirms that the sugar and starch profile in maize is influenced by N treatments, growth stage, and tissue type (Table 2), which is consistent with previous findings (Amoah and Kaiser 2025) and enhances our understanding of N management in maize. Specifically, (i) N treatment induced distinct sucrose and starch pattern across stages, (ii) LN boosted starch in the upper leaves, and (iii) LN plants maintained higher carbohydrates levels than other N treatment plants. These results emphasize that N forms and availability shape carbohydrate partitioning strategies. LN-treated plants retained more carbohydrates, indicating a shift toward energy conservation under stress. Future studies will examine the molecular and enzymatic mechanisms controlling sugar and starch redistribution between source and sink tissues.

## Conclusion

**Fig. 9:**
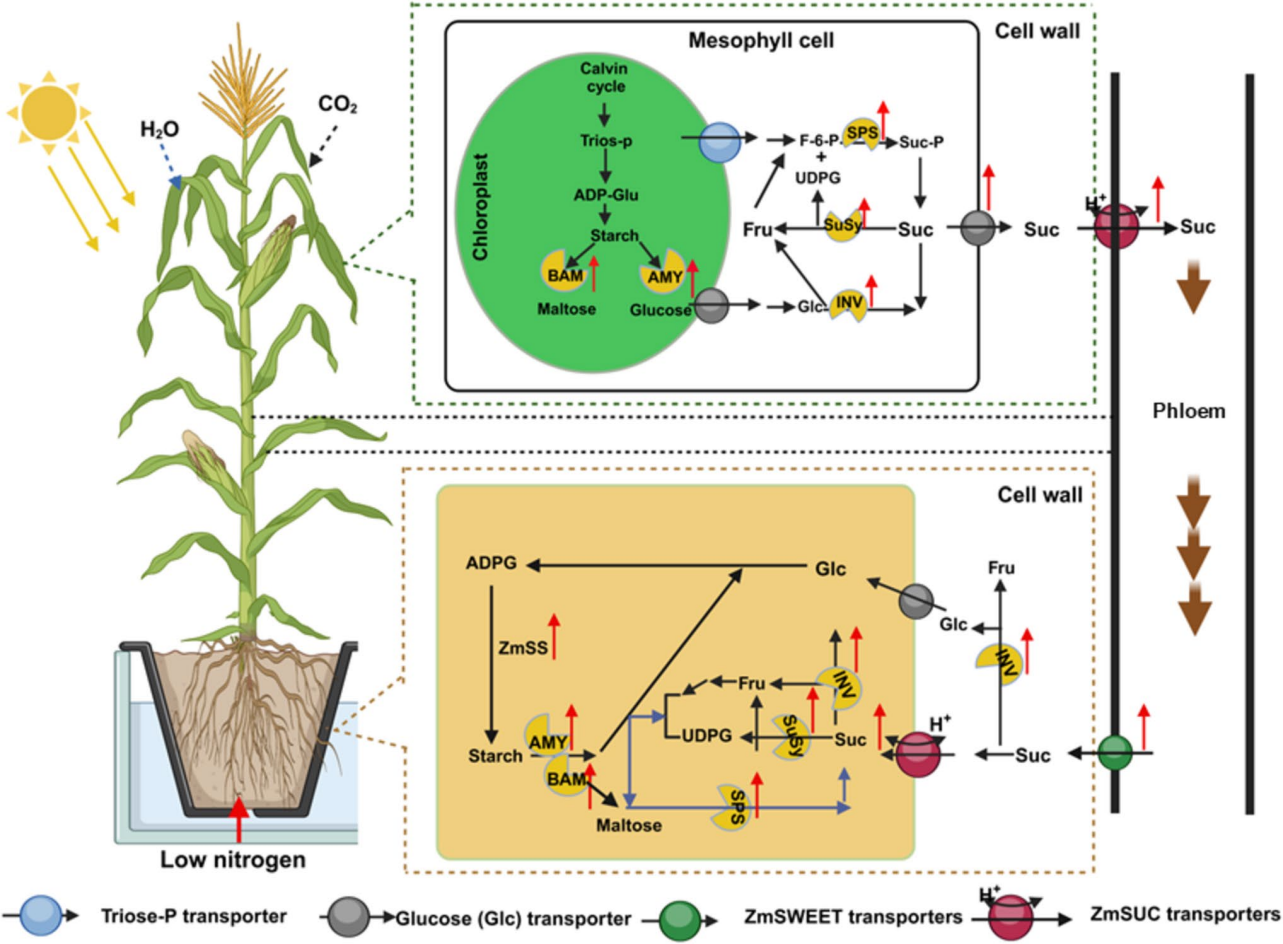
A proposed model of sugar regulation in maize seedlings in response to N deficiency (low nitrogen; LN). LN triggers a sugar-mediated tandem reaction, enhancing maize tolerance to N deficiency. N limitation stress modifies the expression of key regulatory metabolic genes and the activities of sugar metabolism enzymes. This modulation influences sugar accumulation and activates sugar transporter transcription, thereby regulating sugar allocation for environmental adaptation. Upward red arrows represent upregulated components

This study demonstrated that low-nitrogen (LN) treatment increased the root-to-shoot (R/S) ratio in maize seedlings. This increase was attributed to a greater inhibition of shoot biomass accumulation compared to root biomass. Under LN treatment, maize growth was predominantly regulated by sugar metabolism, allocation, and transport (Fig. 9). LN conditions enhanced the activities of sucrose and starch metabolism enzymes and upregulated the expression of genes such as *ZmSPS1*, *ZmSuSy1*, *ZmVINV1*, *ZmCINV1*, *ZmCWINV1*, *ZmAMY1*, *ZmAGPase1*, and *ZmBAM1*. These changes improved the efficiency of sucrose and starch utilization. Furthermore, LN treatment upregulated the expression of sugar transporter genes, including *ZmSWEET14*, *ZmSUC2*, and *ZmSTP2*, in both the leaves and roots of maize seedlings. This facilitated the transport of sucrose from leaves to roots. In summary, nitrogen deficiency elevated the levels of soluble sugars and starch in maize by regulating sugar metabolism and transport. This mechanism appears to be a preferred strategy for sustaining root growth and metabolism under N-limited conditions. Future studies will explore the effects of various N forms through several approaches. (i) Conducting field trials across diverse agricultural soils and environmental conditions would help validate these findings and provide practical insights. (ii) Examining different genotypes and crop species is essential to assess the broader implications for NUE, as the present study focused on the high-performing maize inbred line TX-40 J, which may limit the applicability of the results to other maize varieties and crops. (iii) Incorporating detailed anatomical and transcriptomic analyses would offer deeper insights into the physiological and molecular impacts of N availability on maize growth and development.

## Supplementary Information

Below is the link to the electronic supplementary material.

Supplementary file1 (DOCX 1242 KB)

Supplementary file2 (DOCX 22 KB)

## Data Availability

Data are contained in the manuscript.

## Abbreviations

AMY: α-Amylase
BAM: β-Amylase
DAT: Days after treatment
INV: Invertase (C, cytoplasmic; CW, cell wall; V, vacuolar)
HN–MN–LN: High–medium–low N treatment
LA: Low ammonium treatment
R/S: Root-to-shoot ratio
SPS: Sucrose phosphate synthase
SS: Starch synthase
SuSy: Sucrose synthase
TNC: Total non-structural carbohydrate
