# Supplementary material for: Nitrogen deficiency impacts growth and modulates carbon metabolism in maize
Source: Planta. 2025 Sep 2;262(4):94. doi: 10.1007/s00425-025-04814-x (PMC12405021; doi:10.1007/s00425-025-04814-x)
Supplement: Supplementary file 1 — Supplementary file1 (DOCX 1242 KB) [file 425_2025_4814_MOESM1_ESM.docx]

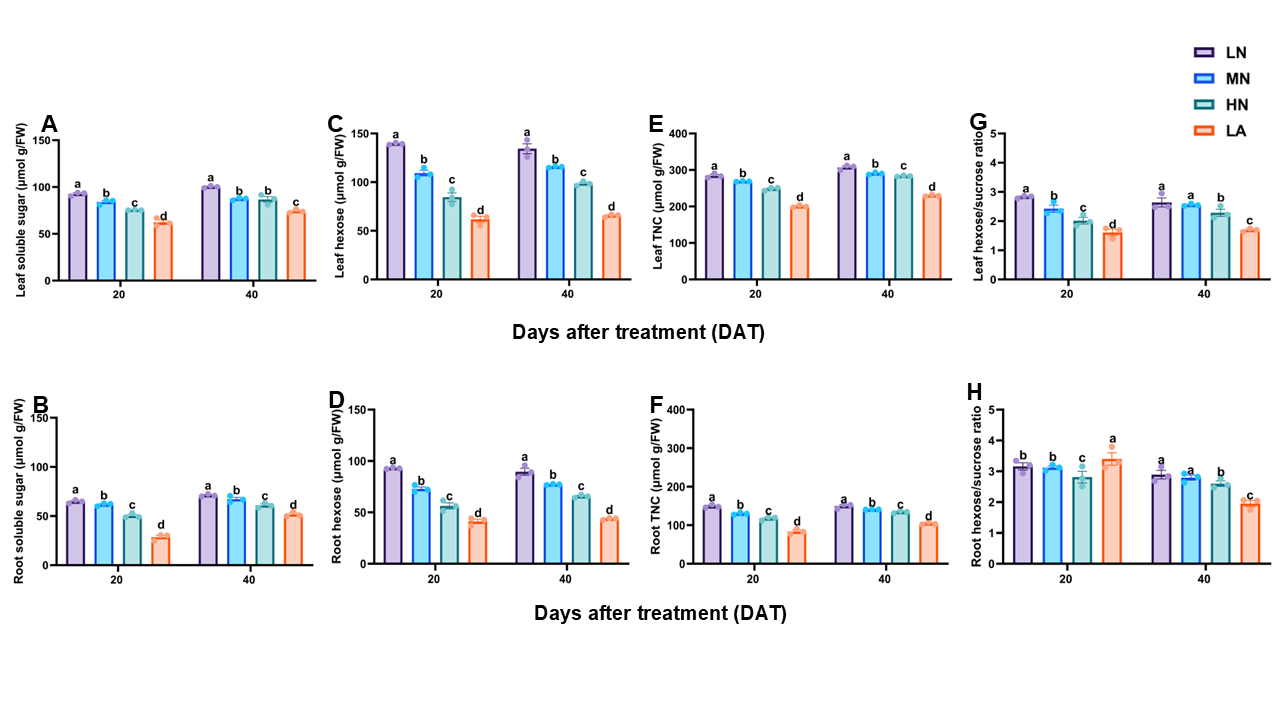


**Fig. S1** Effect of different nitrogen forms on soluble sugar, hexose, total non-structural carbohydrate and hexose: sucrose ratio in the leaves (**A**, **C**, **E**, **G**) and roots (**B**, **D**, **F**, **H**) of maize inbred line TX-40J. Data are presented as mean ± SE (*n* = 6). Statistical significance was determined using Tukey's multiple range test (*P* ≤ 0.05), with different letters indicating significant differences between treatments. FW, fresh weight; LN, low nitrogen (N deficiency); MN, moderate nitrogen; HN, high nitrogen; LA, low ammonium treatment.


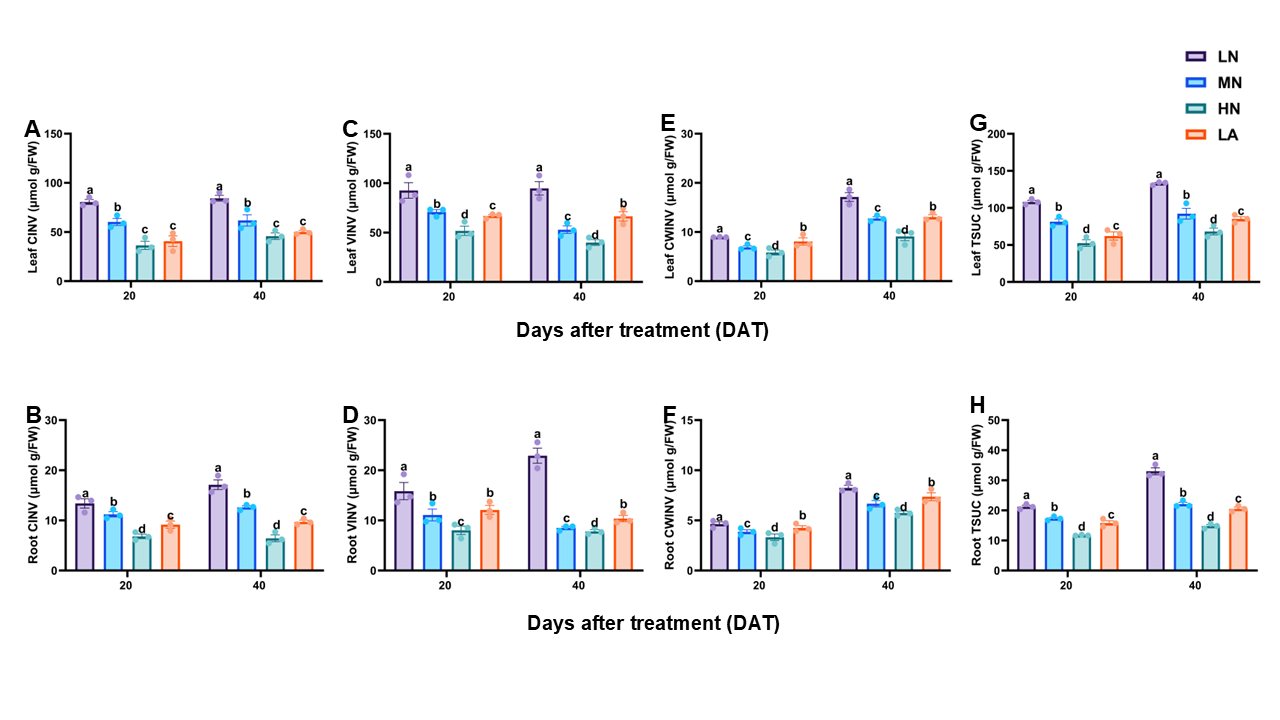


**Fig. S2** Effect of different nitrogen forms on cytoplasmic invertase, vacuolar invertase, cell wall invertase, total sucrolytic activity in the leaves (**A**, **C**, **E**, **G**) and roots (**B**, **D**, **F**, **H**) of maize inbred line TX-40J. Data are presented as mean ± SE (*n* = 6). Statistical significance was determined using Tukey's multiple range test (*P* ≤ 0.05), with different letters indicating significant differences between treatments. FW, fresh weight; LN, low nitrogen (N deficiency); MN, moderate nitrogen; HN, high nitrogen; LA, low ammonium treatment.


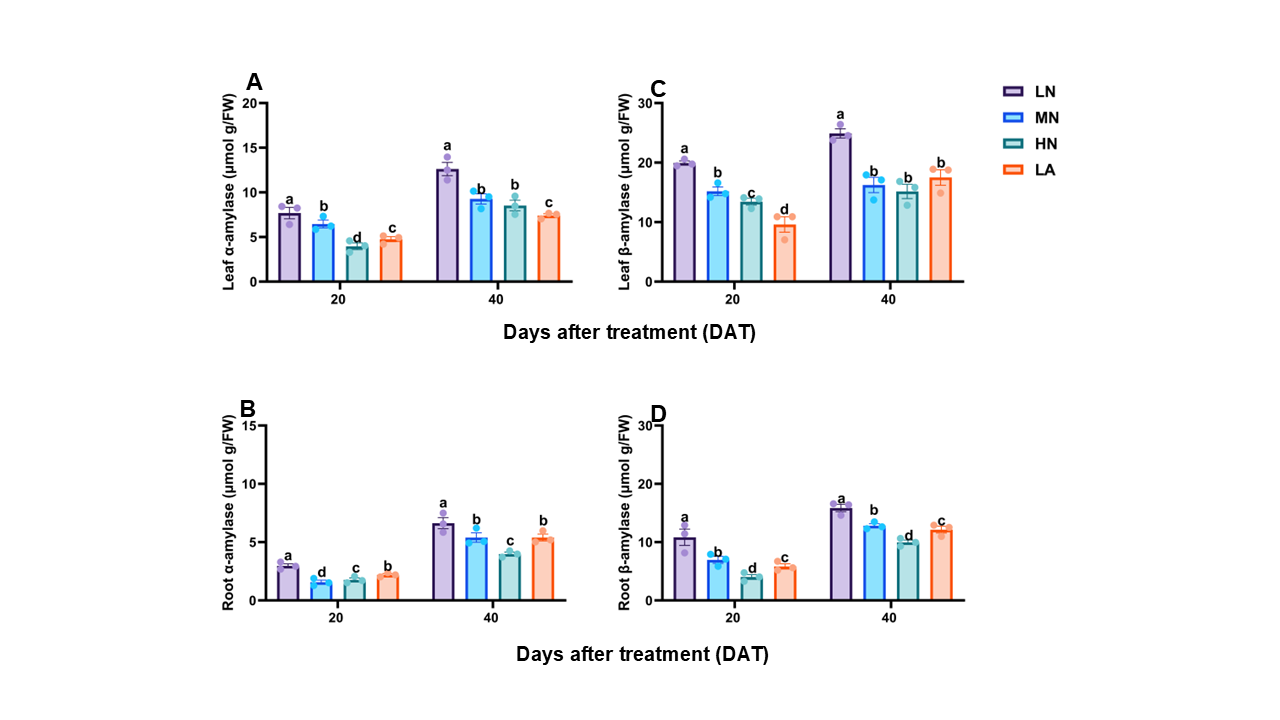


**Fig. S3** Effect of different nitrogen forms on starch metabolizing enzymes activity. Leaf (A) and root (**B**) α-amylase and leaf (**C**) and root (**D**) β-amylase activity. Data are presented as mean ± SE (*n* = 6). Statistical significance was determined using Tukey's multiple range test (*P* ≤ 0.05), with different letters indicating significant differences between treatments. FW, fresh weight; LN, low nitrogen (N deficiency); MN, moderate nitrogen; HN, high nitrogen; LA, low ammonium treatment.


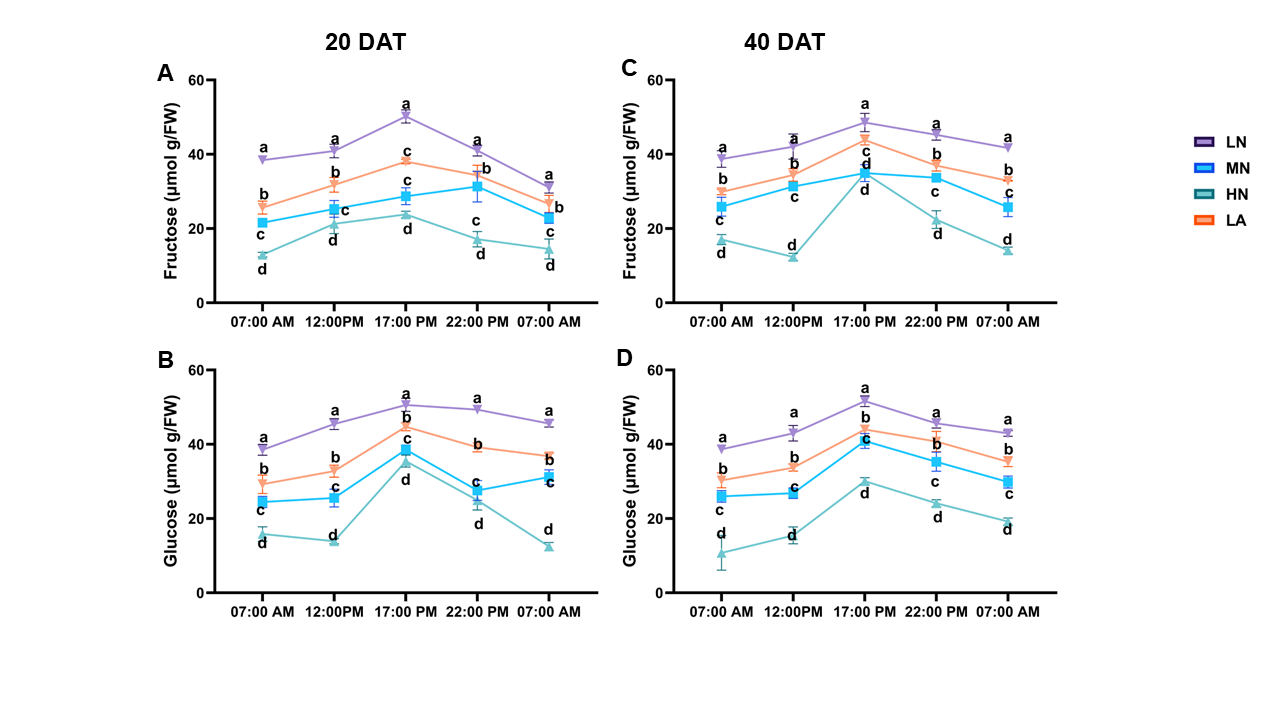


**Fig. S4** Diurnal changes in leaf fructose (**A**) and glucose (**B**) at 20 days after treatment (DAT) and leaf fructose (**C**) and glucose (**D**) at 40 DAT under different nitrogen treatments. Samples were collected at 7:00, 12:00, 17:00, 22:00, and 7:00 on the second day. Data are presented as mean ± SE (*n* = 6). Statistical significance was determined using Tukey's multiple range test (*P* ≤ 0.05), with different letters indicating significant differences between treatments. FW, fresh weight; LN, low nitrogen (N deficiency); MN, moderate nitrogen; HN, high nitrogen; LA, low ammonium treatment.


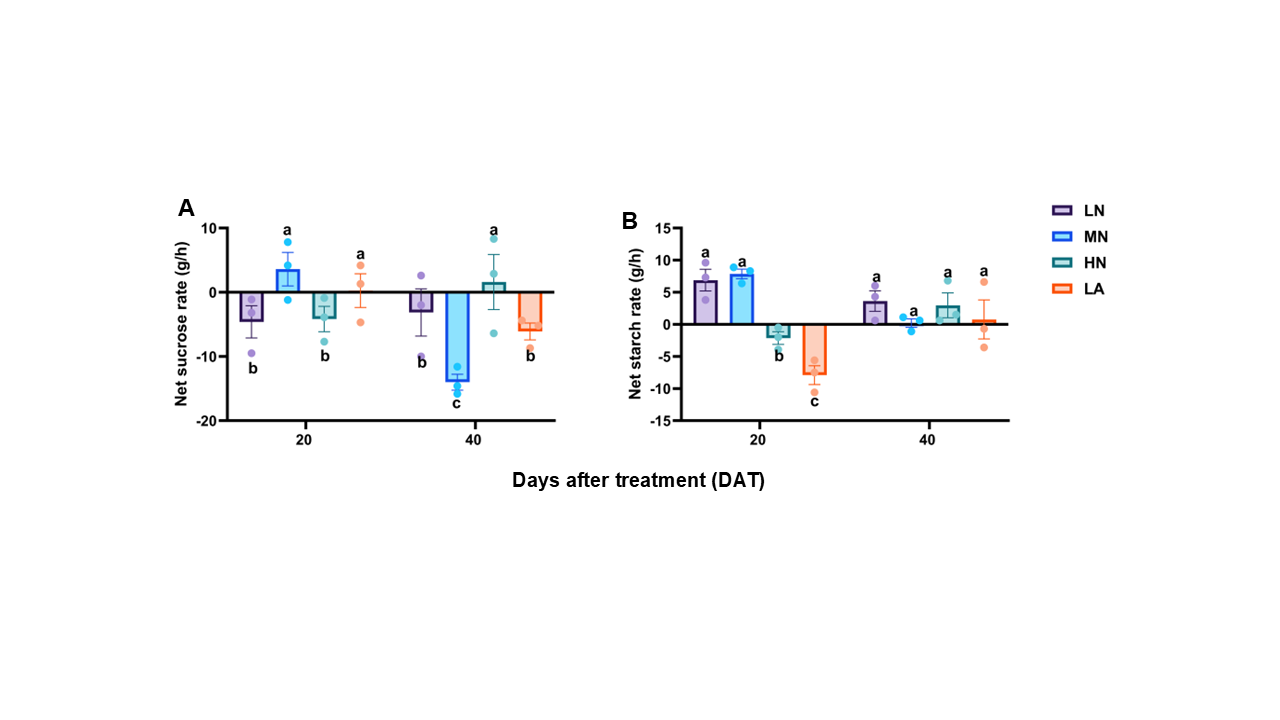


**Fig. S5** Net sucrose (**A**) and net starch (**B**) accumulation rate in maize inbred line TX-40J grown under varying nitrogen treatments. Data are presented as mean ± SE (*n* = 6). Statistical significance was determined using Tukey's multiple range test (*P* ≤ 0.05), with different letters indicating significant differences between treatments. FW, fresh weight; LN, low nitrogen (N deficiency); MN, moderate nitrogen; HN, high nitrogen; LA, low ammonium treatment.


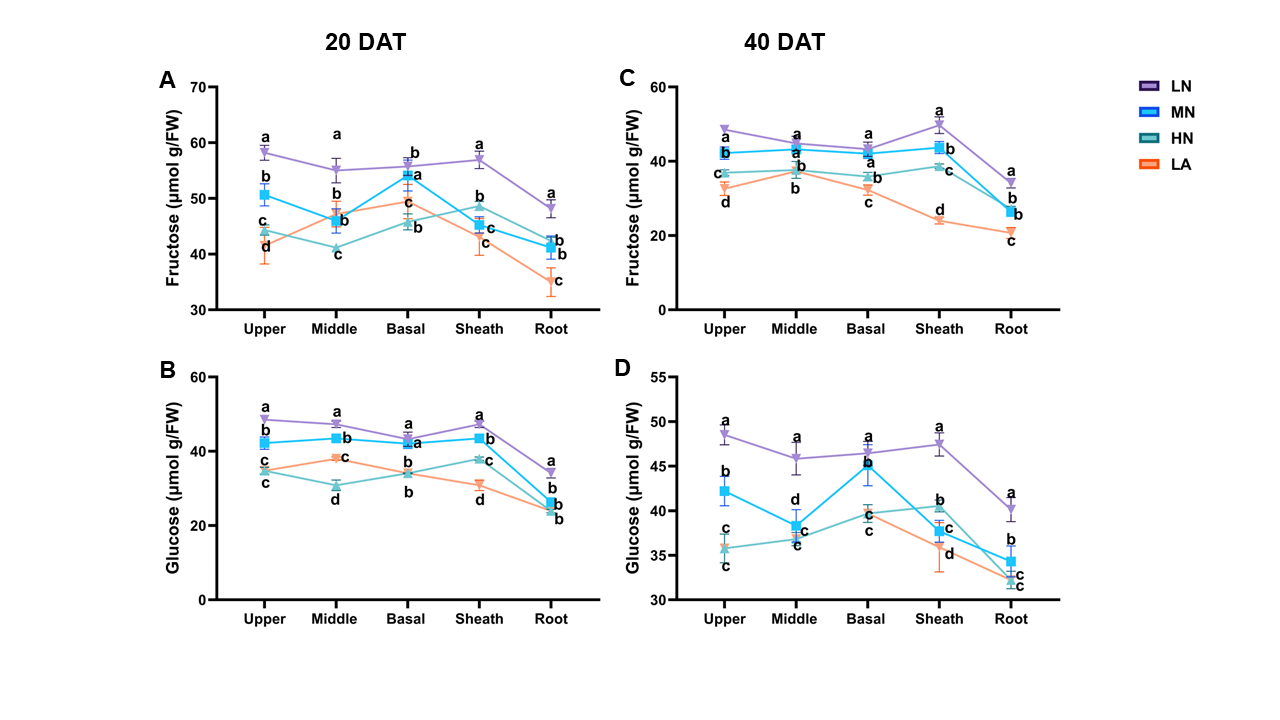


**Fig. S6** Leaf fructose (**A**) and glucose (**B**) at 20 DAT, and leaf fructose (**C**) and glucose (**D**) at 40 DAT in different tissues under various nitrogen form treatments. Data are presented as mean ± SE (*n* = 6). Statistical significance was determined using Tukey's multiple range test (*P* ≤ 0.05), with different letters indicating significant differences between treatments. FW, fresh weight; LN, low nitrogen (N deficiency); MN, moderate nitrogen; HN, high nitrogen; LA, low ammonium treatment.
