# Supplementary material for: Nitrogen deficiency impacts growth and modulates carbon metabolism in maize
Source: Planta. 2025 Sep 2;262(4):94. doi: 10.1007/s00425-025-04814-x (PMC12405021; doi:10.1007/s00425-025-04814-x)
Supplement: Supplementary file 2 — Supplementary file2 (DOCX 22 KB) [file 425_2025_4814_MOESM2_ESM.docx]

**Table S1** Composition of the nutrients used for treatments

| Chemical | Final solution (mM) | | | |
| --- | --- | --- | --- | --- |
|  | **LN (1 mM NO₃⁻)** | **MN (2 mM NO₃⁻)** | **HN (10 mM NO₃⁻)** | **LA ( 1 mM NH₄⁺)** |
| MgSO_4_.7H_2_O  KH_2_PO_4_ | 1.0000  1.0000 | 1.0000  1.0000 | 1.0000  1.0000 | 1.0000  1.0000 |
| Trace elements | | | | |
| H_3_BO_3_  MnSO_4_.H_2_O  ZnSO_4_.7H_2_O  CuSO_4_.5H_2_O  Na_2_MoO_4_.2H_2_O  KCl | 0.0500  0.0050  0.0010  0.0010  0.0007  0.0500 | 0.0500  0.0050  0.0010  0.0010  0.0007  0.0500 | 0.0500  0.0050  0.0010  0.0010  0.0007  0.0500 | 0.0500  0.0050  0.0010  0.0010  0.0007  0.0500 |
| Fe-Stock | | | | |
| Fe-Na-EDTA  FeEDDHA | 0.1000  0.1000 | 0.1000  0.1000 | 0.1000  0.1000 | 0.1000  0.1000 |
| N Stocks | | | | |
| Ca(NO_3_)_2_.4H_2_O  KNO_3_  (NH₄)₂SO₄ | 0.2500  0.5000  0.0000 | 0.5000  1.0000  0.0000 | 2.5000  5.0000  0.0000 | 0.0000  0.0000  1.0000 |
| K & Ca (supplement as required ) | | | | |
| K_2_SO_4_  CaCl_2_.2H_2_O  CaSO_4_.2H_2_O  KCl | 0.2500  0.2500  1.7500  0.1000 | 0.2500  0.2500  1.7500  0.1000 | 0.2500  0.2500  1.7500  0.1000 | 2.8000  0.0000  4.0000  0.0000 |

**Table S2** List of equations used for estimating sucrose and starch synthesis, degradation and net accumulations rates

**1. Sucrose and starch degradation equations:**

Sucrose synthesis rate = $\frac{Sucrose at 12:00 PM-Sucrose at 07:00 AM}{5h}$ (1)

Starch synthesis rate = $\frac{Starch at 12:00 PM-Starch at 07:00 AM}{5h}$ (2)

**2. Sucrose and starch degradation equations:**

Sucrose degradation rate = $\frac{Sucrose at 10:00 PM-Sucrose at 07:00 AM (Next day)}{9 h}$ (3)

Starch degradation rate = $\frac{Starch at 10:00 PM-Starch at 07:00 AM (Next day)}{9 h}$ (4)

**3. Net sucrose and starch accumulation rates:**

Net sucrose accumulation = Sucrose synthesis value – Sucrose degradation value (5)

Net starch accumulation = Starch synthesis – Starch degradation values (6)

**Table S3** List of primers used for quantitative polymerase chain reaction analyses

| NCBI LOCs | Gene name | 5' - 3' (Forward primer) | 3' - 5' (Reverse primer) |
| --- | --- | --- | --- |
| LOC542247  LOC542711  LOC541615  LOC542737  LOC541669  LOC100273083  LOC100280251  LOC100273029  LOC542472  LOC542324  LOC100382511  J01238.1  LOC541665  LOC542314 | *ZmSuSy*  *ZmSPS*  *ZmSUC2*  *ZmAGPase*  *ZmSS*  *ZmSTP2*  *ZmSUT2*  *ZmAMY1*  *ZmBAM1*  *ZmVINV*  *ZMCINV*  *ZmActin*  *ZmUBQc*  *ZmCWINV* | ACCCATCCATTCCACCTCCG  CCAGCGGCATGTGAATTTGA  CCTCACTACCCGCGCTCTC  GCGCAATCGATCCATCCGTC  GAGGAGGCATGCCCTTGACA  CTCCTCTCGTCGTGGTTCAC  TGGGATGCGAGAGGAAACGG  TGTACACGGGACTGCATTGAT  GCTGAGCTCACTGCCGGATA  CACTCTGCACCGCAACAAAT  GAAGCAACTGCAGAAATGGGC  GCCTTGACCATGTTGGGTGTG  CCTGCTCTCCATCTGCTCCC  TGACCGCCTGGGAGATGAAG | CACCTGCACCTTCCCCCATT  AGCATACAATCTTGACAGTCGTA  TGTGATCGAAATCGAAGGGGAT  ACCTCCACTGCCTCTCCTCA  CATTGAGGCCCTGTCCACCT  AAAAGCACAGCCTGATTCGC  CTGTGCGTCCGTCCTGTTTG  TATGCATGGCTCCAATTGCC  CCTCAGGTGCACTTTTCGCC  AGAGGCTACACGTCTTCCCA  AGCACTTATGCTTCACAGGGA  TCCAAATGGGCCCTGAGAGA  ATCGCGTACTTCTGCGTCCA  TCTCCTTTGGCGGCAGAGAC |
